# Secondary Metabolites of the Genus *Didemnum*: A Comprehensive Review of Chemical Diversity and Pharmacological Properties

**DOI:** 10.3390/md18060307

**Published:** 2020-06-11

**Authors:** Diaa T. A. Youssef, Hadeel Almagthali, Lamiaa A. Shaala, Eric W. Schmidt

**Affiliations:** 1 Department of Natural Products, Faculty of Pharmacy, King Abdulaziz University, Jeddah 21589, Saudi Arabia; halmagthali@stu.kau.edu.sa; 2 Department of Pharmacognosy, Faculty of Pharmacy, Suez Canal University, Ismailia 41522, Egypt; 3 Department of Pharmacognosy, College of Pharmacy, Taif University, Al-Haweiah 21974, Saudi Arabia; 4 Natural Products Unit, King Fahd Medical Research Center, King Abdulaziz University, Jeddah 21589, Saudi Arabia; lshalla@kau.edu.sa; 5 Department of Medical Laboratory Technology, Faculty of Applied Medical Sciences, King Abdulaziz University, Jeddah 21589, Saudi Arabia; 6 Suez Canal University Hospital, Suez Canal University, Ismailia 41522, Egypt; 7 Department of Medicinal Chemistry, University of Utah, Salt Lake City, UT 84112, USA

**Keywords:** tunicate, ascidian, genus *Didemnum*, secondary metabolite, chemical diversity, natural product, biological properties

## Abstract

Tunicates (ascidians) are common marine invertebrates that are an exceptionally important source of natural products with biomedical and pharmaceutical applications, including compounds that are used clinically in cancers. Among tunicates, the genus *Didemnum* is important because it includes the most species, and it belongs to the most speciose family (Didemnidae). The genus *Didemnum* includes the species *D.* *molle*, *D.* *chartaceum*, *D.* *albopunctatum*, and *D.* *obscurum*, as well as others, which are well known for their chemically diverse secondary metabolites. To date, investigators have reported secondary metabolites, usually including bioactivity data, for at least 69 members of the genus *Didemnum*, leading to isolation of 212 compounds. Many of these compounds exhibit valuable biological activities in assays targeting cancers, bacteria, fungi, viruses, protozoans, and the central nervous system. This review highlights compounds isolated from genus *Didemnum* through December 2019. Chemical diversity, pharmacological activities, geographical locations, and applied chemical methods are described.

## 1. Introduction

**Figure 1:**
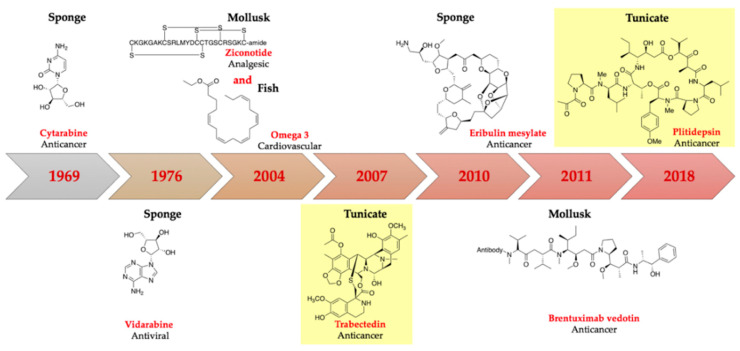
Sample of approved marine natural products. Those in yellow highlight originate in tunicates.

The biodiversity of organisms in the marine ecosystem has motivated researchers to discover many marine natural products that might ultimately be developed into therapeutics [1]. Among marine organisms, invertebrates such as ascidians, sponges, molluscs, and bryozoans represent the major source of organic extracts with significant bioactivities [2]. Currently, there are eight marine-derived drugs approved by different agencies, including the U.S. FDA, European Medicines Agency (EMEA), Japanese Ministry of Health, and Australia’s Therapeutic Goods Administration (Figure 1). An additional 22 drug leads are currently in different phases (I–III) of drug development [3]. Considering that ≈1000 new molecules have been isolated from marine organisms annually for the past few years, the biotechnological and pharmaceutical potential of the sea remains awe-inspiring [4,5,6,7,8,9]. 

**Figure 2:**
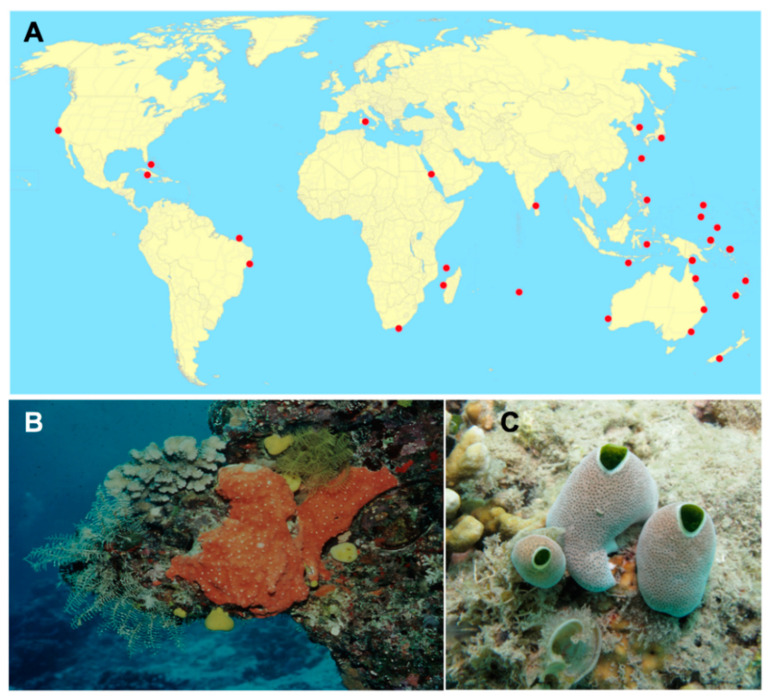
The genus *Didemnum* and relatives. (**A**) A map with red dots indicating collection locations for *Didemnum* spp. described in this review. (**B**) An orange colony exhibiting sheet-like growth common among *Didemnum* spp. tunicates. This specific species is not *Didemnum*, but instead the same *Polysyncraton lithostrotum* specimen from which namenamicin (**129**) was originally isolated. Compound **129** was also later isolated from *Didemnum* sp. (**C**) The ubiquitous tropical tunicate, *Didemnum molle*, showing three individual colonies in a common vase-like morphology found in many *Didemnum* spp. tunicates. This is the same specimen from which divamide A (**190**) was originally isolated. Photos by Chris Ireland, used with permission.

Of the marine invertebrates commonly investigated for marine natural products, as members of Phylum Chordata tunicates (ascidians) are the most closely related to humans. Ascidians represent the most diverse and biggest class of the sub-phylum Tunicata, comprising about 3000 described species [10]. Didemnidae is the largest tunicate family [10], and it has been confirmed to be monophyletic using molecular methods [11]. Didemnidae features many genera that are prolific and famous producers of bioactive natural products, including *Diplosoma*, *Lissoclinum*, *Polysyncraton*, and *Trididemnum* (Figure 2). Among tunicates from family Didemnidae, the genus *Didemnum* stands out, with more described species than any other tunicate [11]. In addition, the number of described species is certainly an underestimate of the true biodiversity of genus *Didemnum*. For example [12], the widespread tunicate *Didemnum molle* exists in a variety of overlapping color morphs. These morphs are genetically monophyletic and deeply divergent, indicating that they are likely to be different species [12]. Further complicating this variety, tunicates from family Didemnidae are colonial, with up to thousands of individual animals known as zooids sharing a single tunic. The individual colonies are often comprised of hybrids, or mixtures, of genetically different zooids that have mixed by colony fusion [13]. Finally, the genus *Didemnum* harbors many different symbiotic bacteria, which are sometimes responsible for producing the bioactive secondary metabolites isolated from the whole animals [14]. As a result of these factors, the genus *Didemnum* provides an amazing array of biological and chemical diversity. 

**Figure 3:**
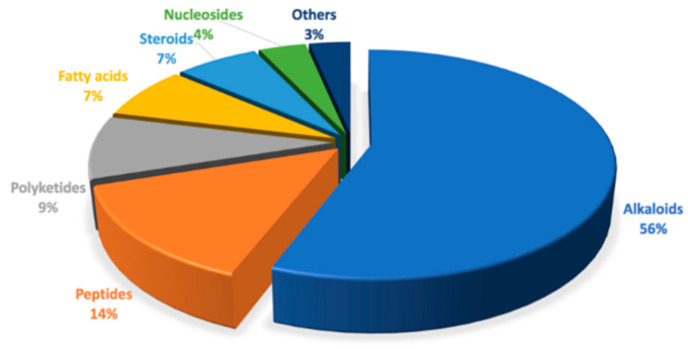
Major compound classes found in *Didemnum* spp. (*n* = 212 as of December 2019).

Tunicates are a vital source of bioactive compounds with promising potential for biomedical applications, including several approved drugs. The production of active compounds in tunicates is thought to result from competition in the marine environment, especially to protect the sedentary animals from predation [15]. As the most speciose genus, *Didemnum* is also very rich in bioactive secondary metabolites [10]. While numerous chemical and biological studies investigate the genus *Didemnum*, most of these studies do not identify the animals to species. These studies show that the genus *Didemnum* is abundant in many classes of natural products, including peptides, alkaloids, indole/alkaloids, β-carboline alkaloids, spiroketals, polyketides, halogenated compounds, steroids, and many others (Figure 3). Biological investigations of these entities have shown that some of these compounds possess anticancer, antimicrobial, and antimalarial activity [15]. 

This review focuses on the diversity of the chemical structures isolated from genus *Didemnum*, the geographical locations of investigated species and, whenever applicable, methods of isolation, spectroscopic methods, and reported biological activities (Tables S1–S6). Searches were performed in SciFinder using the terms “*Didemnum*”, and “Didemnid”. A total of 413 hits, with some duplications, were found. Only papers with reports about isolation of novel secondary metabolites are considered in this review. Other reports, including synthetic, biosynthetic studies, screening of extracts, and ecological and environmental studies, are touched upon but are not described comprehensively. The first report of chemistry from genus *Didemnum* was published in 1981 [16]. Between then and December 2019, we found a total of 212 secondary metabolites reported from at least 69 species belonging to the genus *Didemnum*.

## 2. Secondary Metabolites with No Currently Reported Bioactivity from the Genus *Didemnum* (Table S6)

**Figure 4:**
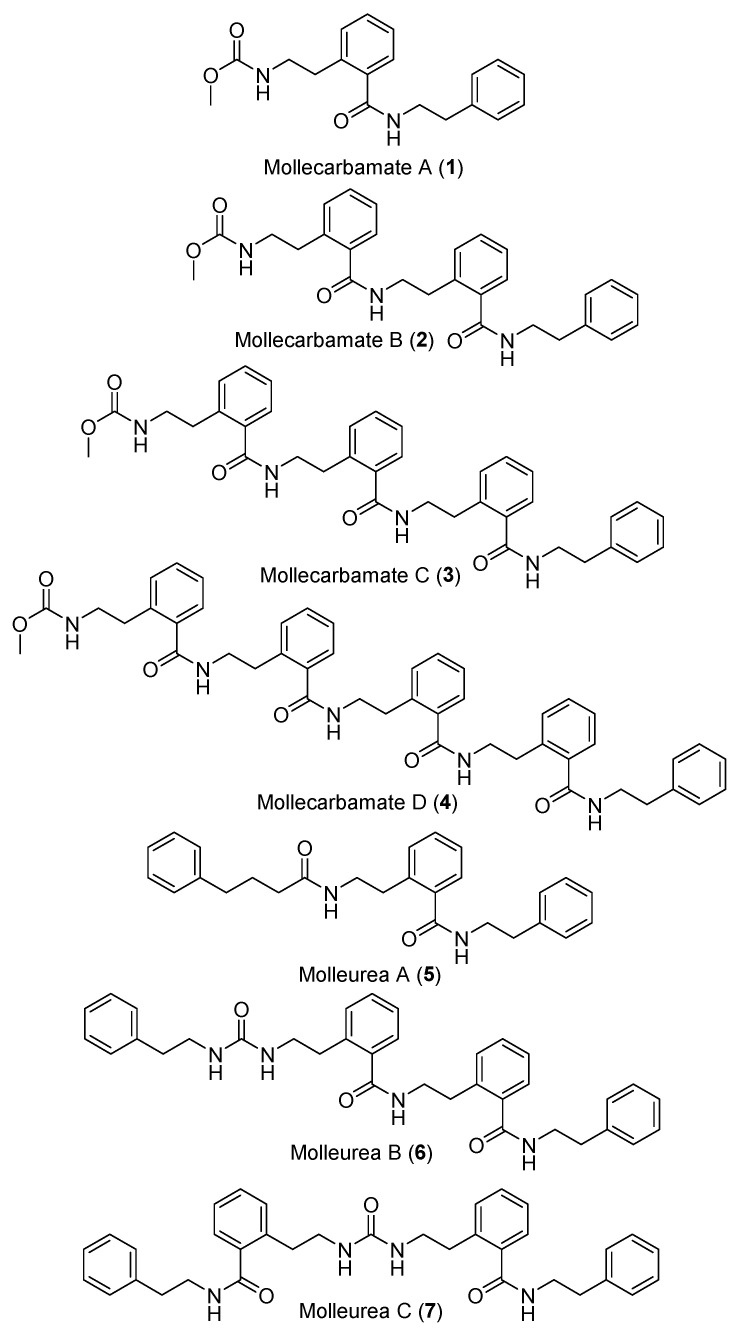
Chemical structures of compounds **1**–**7**.

**Figure 5:**
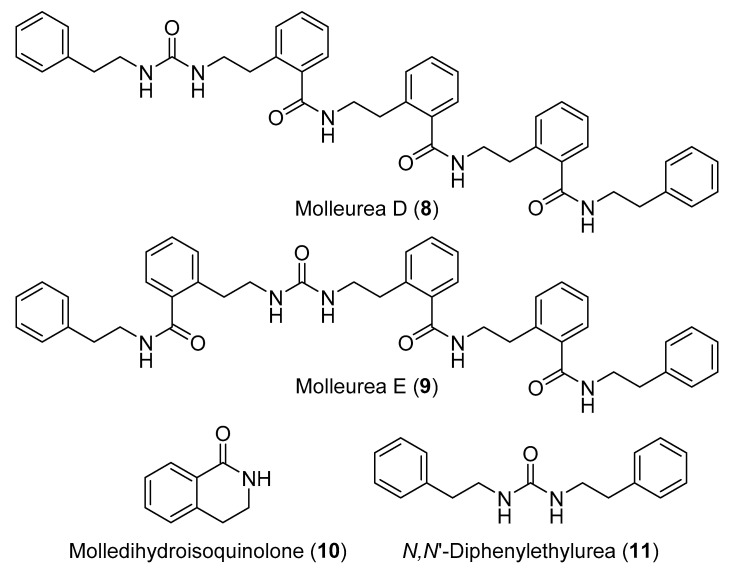
Chemical structures of compounds **8**–**11**.

**Figure 6:**
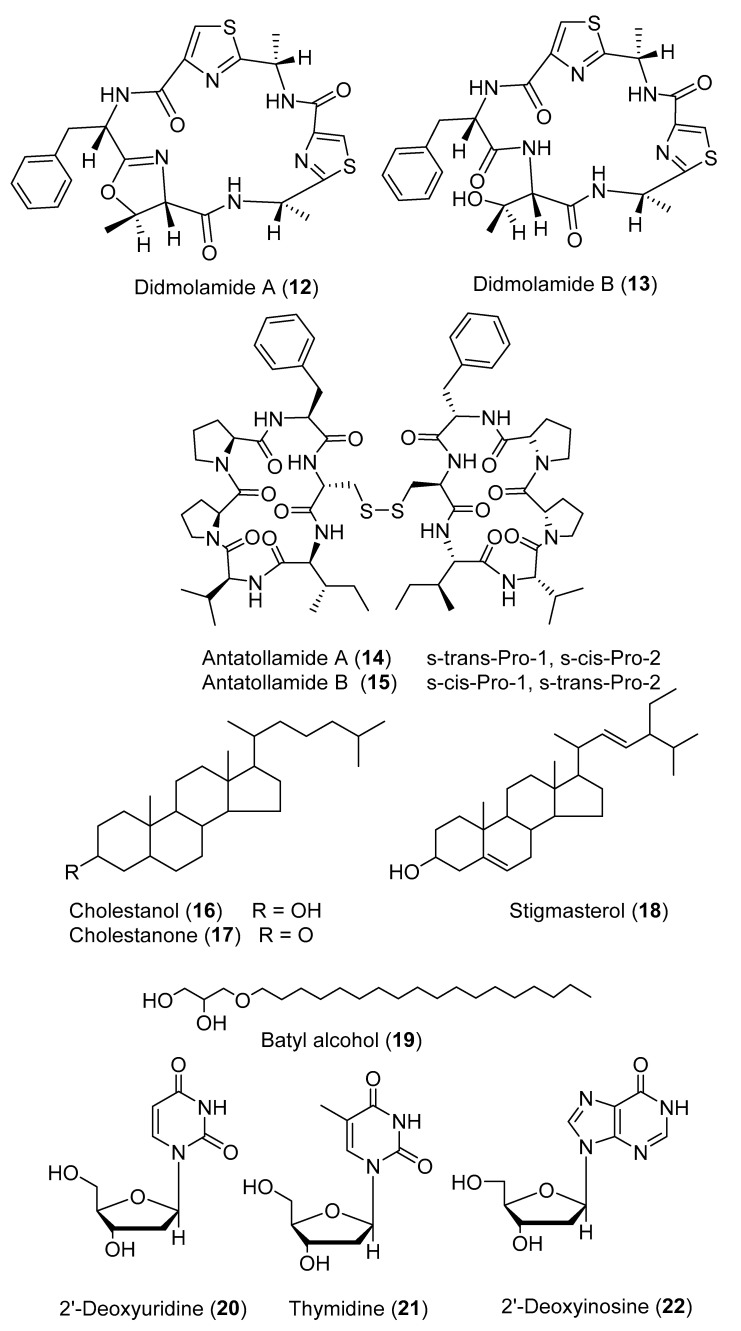
Chemical structures of compounds **12**–**22**.

Eleven compounds were reported from the extracts of two specimens of *D. molle* collected in Madagascar: mollecarbamates A–D (**1**–**4**), molleureas A−E (**5**–**9**), molledihydroisoquinolone (**10**), and *N,N*′-diphenylethylurea (**11**) (Figure 4 and Figure 5). Compounds **1**–**4** and **6**–**10** had no significant antibacterial or anti-HIV activities [17]. The cyclic hexapeptides, didmolamides A and B (**12** and **13**) (Figure 6), were reported from another specimen of *D. molle* collected in Madagascar [18]. 

**Figure 7:**
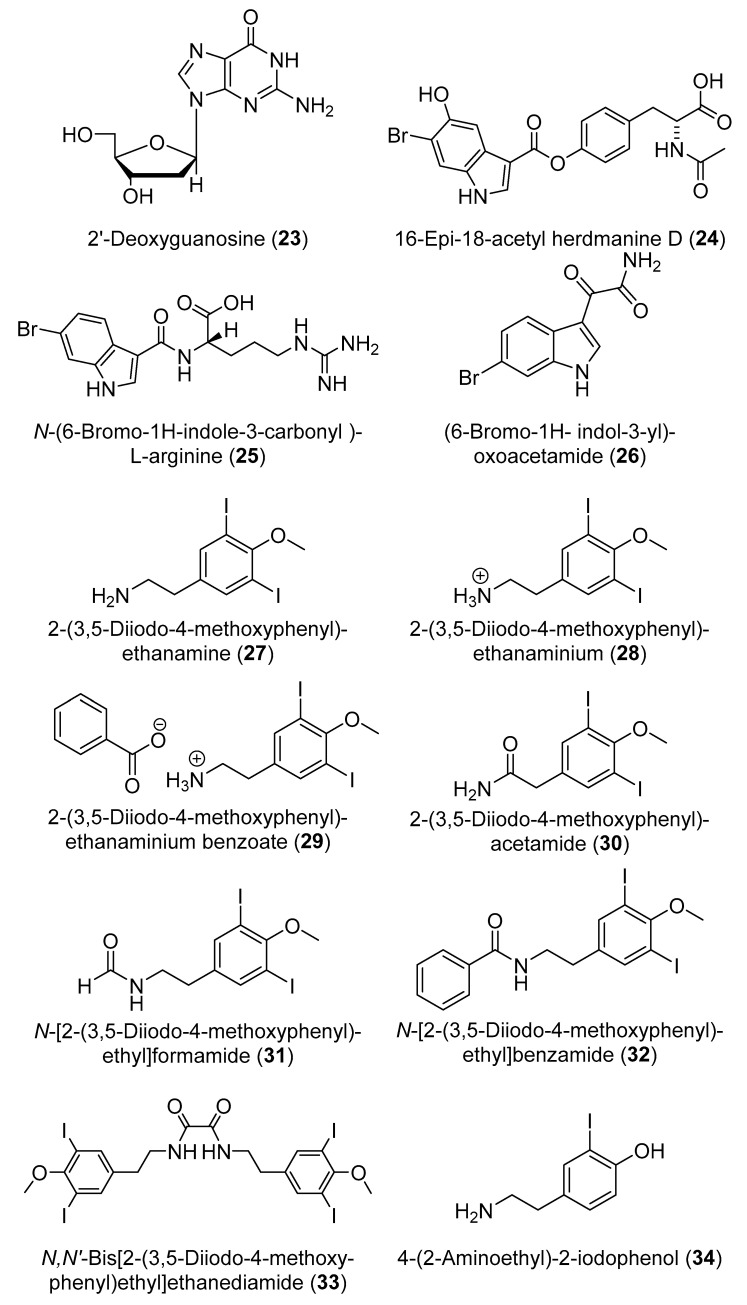
Chemical structures of compounds **23**–**34**.

The dimerized cyclic hexapeptides, antatollamides A and B (**14** and **15**) (Figure 6), were purified from *D. molle* collected in Pohnpei [19]. Three steroids including cholestanol (**16**), a mixture of cholestanone and stigmasterol (**17** and **18**) and batyl alcohol (**19**) (Figure 6) were obtained from *D. psammatodes*, along with the nucleosides 2′-deoxyuridine (**20**), thymidine (**21**), 2′-deoxyinosine (**22**) (Figure 6), and 2′-deoxyguanosine (**23**) (Figure 7) [20]. A group of indole alkaloids including 16-*epi*-18-acetyl herdmanine D (**24**), *N*-(6-bromo-1*H*-indole-3-carbonyl)-l-arginine (**25**), and (6-bromo-1*H*-indol-3-yl)oxoacetamide (**26**) (Figure 7), were reported from a Korean *Didemnum* sp. [21]. 

**Figure 8:**
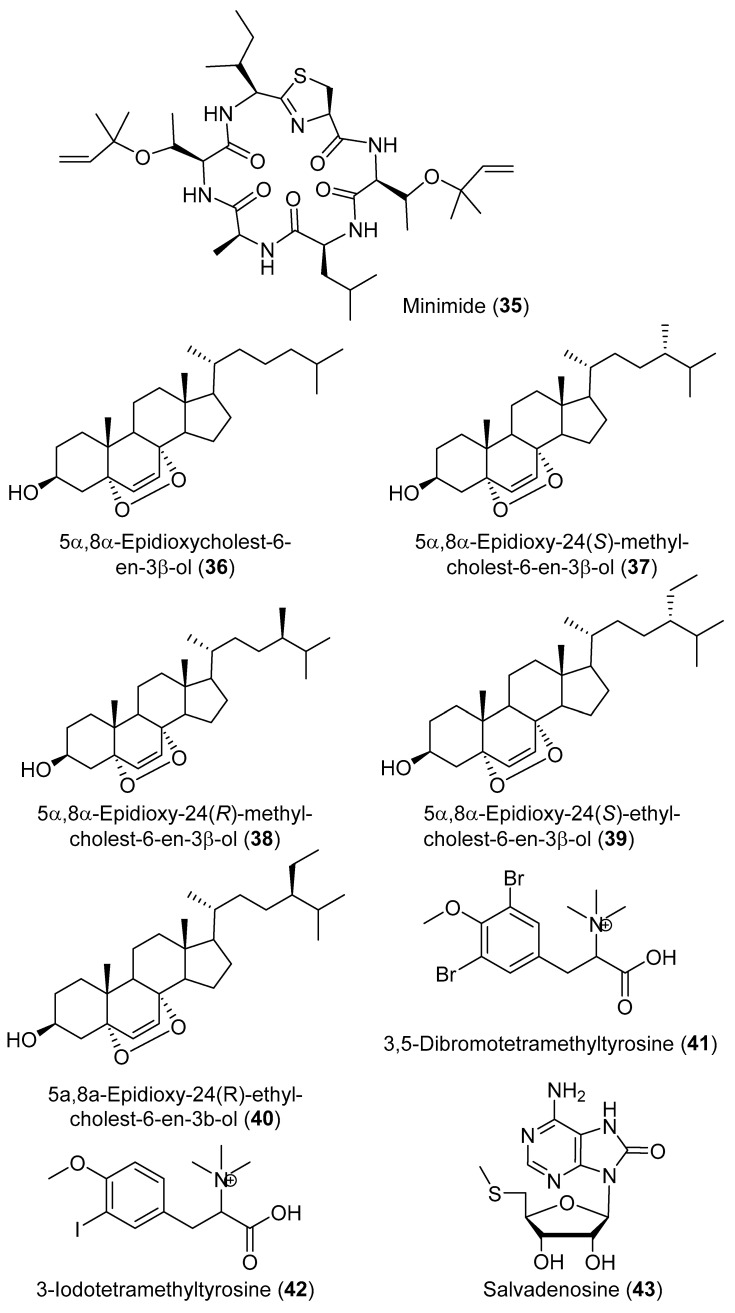
Chemical structures of compounds **35**–**43**.

2-(3,5-Diiodo-4-methoxyphenyl)ethanamine (**27**) (Figure 7) is an iodinated tyramine derivative which forms one of the most common moieties in compounds isolated from diverse specimens of the genus *Didemnum* [21]. Chemical investigation of an aqueous extract of *D. rubeum* resulted in the identification of a series of iodinated compounds derived from compound **27** including 2-(3,5-diiodo-4-methoxyphenyl)ethanaminium (**28**), 2-(3,5-diiodo-4-methoxyphenyl)ethanaminium benzoate (**29**), 2-(3,5-diiodo-4-methoxyphenyl)acetamide (**30**), *N*-[2-(3,5-diiodo-4-methoxyphenyl)ethyl]formamide (**31**), *N*-[2-(3,5-diiodo-4-methoxyphenyl)ethyl]benzamide (**32**), *N,N*′-bis[2-(3,5-diiodo-4-methoxyphenyl)ethyl]ethanediamide (**33**), along with 4-(2-aminoethyl)-2-iodophenol (**34**) (Figure 7) [22]. A cyclic peptide, minimide (**35**) (Figure 8), was discovered by genome mining the symbiotic *Prochloron* bacteria living in *D. molle*, synthesized in *Escherichia coli*, and then found to be identical to the major natural products in the whole organic extracts of the same *D. molle* from the Solomon Islands [23].

**Figure 9:**
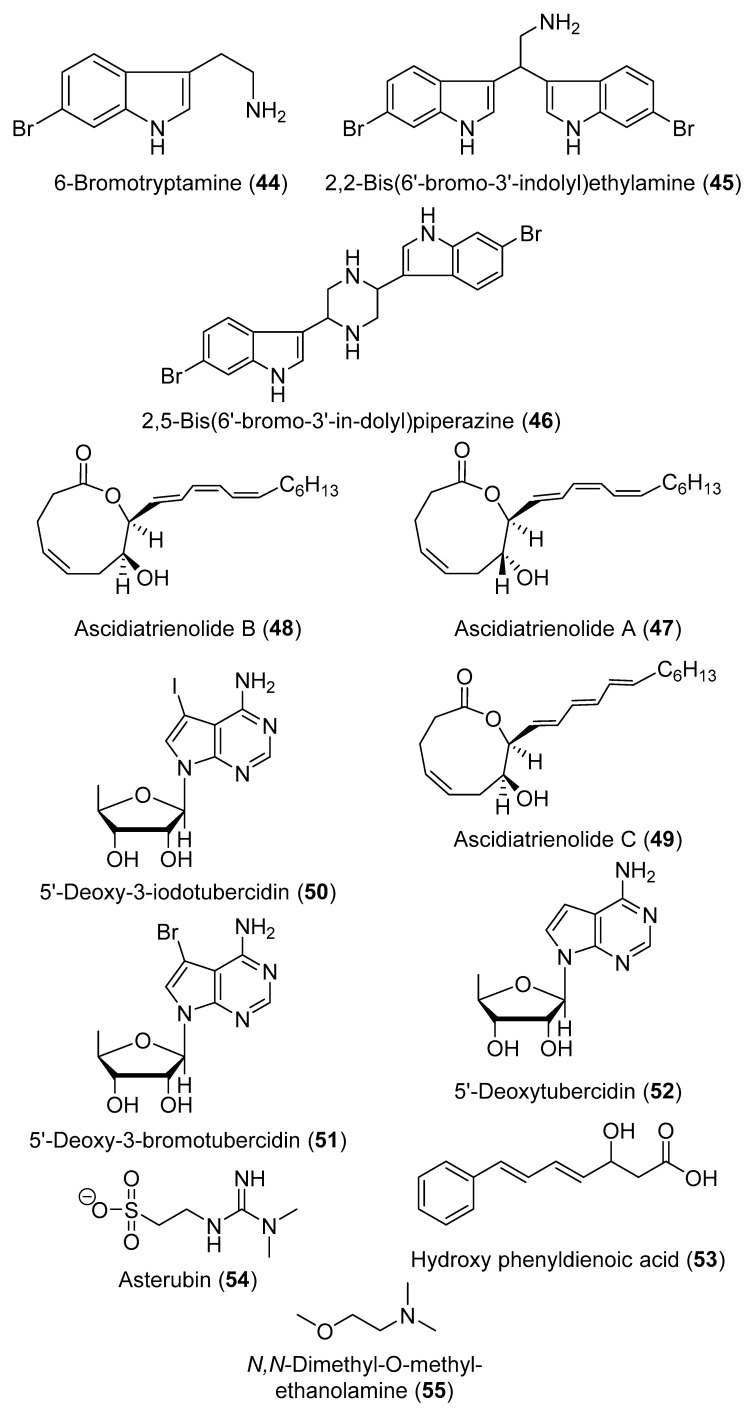
Chemical structures of compounds **44**–**55**.

Five 5α,8α-epidioxysterols derivatives were isolated from *D. salary* including 5α,8α-epidioxycholest-6-en-3β-ol (**36**), 5α,8α-epidioxy-24(*S*)-methylcholest-6-en-3β-ol (**37**), 5α,8α-epidioxy-24(*R*)-methylcholest-6-en-3β-ol (**38**), 5α,8α-epidioxy-24(*S*)-ethylcholest-6-en-3β-ol (**39**), and 5α,8α-epidioxy-24(*R*)-ethylcholest-6-en-3β-ol (**40**) [24]. Bromley and co-authors have reported two halogenated compounds from a South African unidentified *Didemnum* sp., namely 3,5-dibromotetramethyltyrosine (**41**) and 3-iodotetramethyltyrosine (**42**) (Figure 8) [25]. Salvadenosine (**43**) (Figure 8), an uncommon 5′-deoxy-5′-(methylthio) nucleoside, was isolated from the Bahaman tunicate *Didemnum* sp., together with 6-bromotryptamine (**44**) (Figure 9) [26]. Moreover, 6-bromotryptamine derivatives were isolated from two specimens of *D. candidum* collected in the southern Gulf of California. 6-Bromotryptamine (**44**) was isolated from the first specimen, whilst 2,2-bis(6′-bromo-3′-indoly1)ethylamine (**45**) and 2,5-bis(6′-bromo-3′-in-doly1)piperazine (**46**) (Figure 9) were reported from the second specimen [27]. Three novel eicosanoids were isolated from *D. candidum* including ascidiatrienolides A–C (**47**–**49**) (Figure 9) [28]. The tubercidin analogs 5′-deoxy-3-iodotubercidin (**50**), 5′-deoxy-3-bromotubercidin (**51**), and 5′-deoxytubercidin (**52**) (Figure 9) were isolated through chemical investigation of *D. voeltzkowi* [29]. Furthermore, hydroxy phenyldienoic acid (**53**) (Figure 9) was identified from the ethanolic extract of *D. granulatum* [30]. Asterubin (**54**) and *N,N*-dimethyl-*O*-methylethanolamine (**55**) (Figure 9) were isolated from *D. ligulum* [31].

## 3. Secondary Metabolites with Reported Biological Activities

### 3.1. Compounds with Antitumor/Anticancer and Antiproliferative Activities (Table S1)

**Figure 10:**
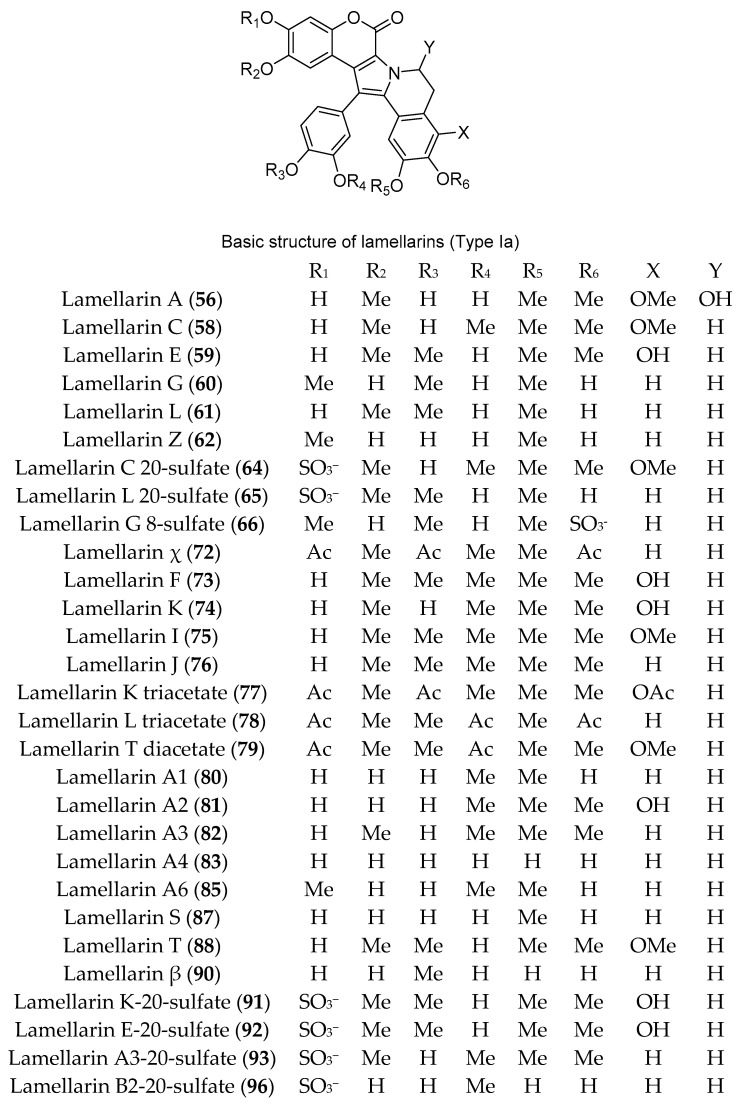
Chemical structures of compounds **56**, **85**–**62**, **64**–**66**, **72**–**83**, **85**, **87**, **88**, **90**–**93** and **96**.

**Figure 11:**
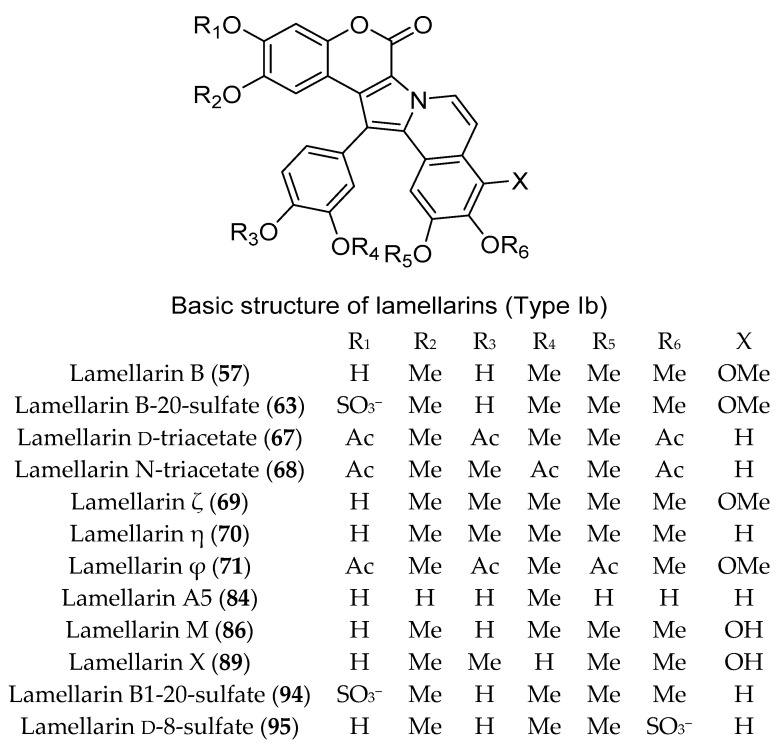
Chemical structures of compounds **57**, **63**, **67**, **68**–**71**, **84**, **94** and **95**.

Numerous DOPA-derived pyrrole alkaloids have been reported from different members of the genus *Didemnum*, including the lamellarins. In 1999, lamellarins A, B, C, E, G, L, and Z (**56**–**62**) (Figure 10 and Figure 11) were isolated from the Australian *D. chartaceum*, along with 20-sulfated derivatives of lamellarins B, C, and L (**63**–**65**) (Figure 10 and Figure 11), the 8-sulfated derivative of lamellarin G (**66**) (Figure 10) and the triacetyl derivatives of lamellarins D and N (**67** and **68**) (Figure 11) [32]. Additionally, chemical analysis of *D. obscurum* resulted in the purification of four new lamellarin alkaloids, Lamellarin-ζ (**69**), lamellarin-η (**70**), lamellarin-φ (**71**), and lamellarin-χ (**72**), together with seven known lamellarins, lamellarins F, K, I, and J (**73**–**76**), lamellarin-K triacetate (**77**), lamellarin-L triacetate (**78**), and lamellarin-T diacetate (**79**) (Figure 10 and Figure 11). In an MTT assay, compounds **68**, **71, 75**, and **76** exhibited excellent inhibition of cell viability towards colorectal cancer cells (COLO-205) with IC_50_ values of 0.0056, 0.0002, 0.00025, and 0.009 μM, respectively [33]. Lamellarins A1–A6 (**80**–**85**) along with lamellarins C (**58**), E (**59**), G (**60**), K (**74**), M (**86**), S (**87**), T (**88**), X (**89**), Z (**62**), and χ (**72**) (Figure 10 and Figure 11) were isolated during analysis of two southern Australian *Didemnum* sp. [34,35,36]. Compounds **80**, **81**, and **87** showed cytotoxic activities towards human colon adenocarcinoma cell line and were P-glycoprotein substrates, while compounds **74**, **59**, **86**, **82**, **58**, and **485** were P-glycoprotein inhibitors and were capable of reversing multi-drug resistance [34]. Lamellarins E (**59**), K (**74**), and M (**86**) displayed antibacterial activity against *Bacillus subtilis* (ATCC 6633) with MIC of 7.5, 15, and 7.5 µM, respectively [34]. Furthermore, when evaluated for their activity against the neurodegenerative disease targets casein kinase 1 (CK1d) and cyclindependent kinase 5 (CDK5), lamellarins A4 (**83**) and S (**87**) displayed high activity with IC_50_ of 3 µM. On the other hand, lamellarins A1 (**80**), A6 (**85**), and Z (**62**) displayed a submicromolar inhibition with IC_50_ of 0.1, 0.3, and 0.4 µM against CDK5, respectively [34]. Lamellarins L (**61**), K (**74**), and I (**75**) showed similar and significant cytotoxicity against P388 and A549 cell lines in culture with IC_50_ values of 0.48, 0.45, and 0.44 µM against each cell line [35]. Further, lamellarins L (**61**) and K (**74**) also exhibited moderate immunomodulatory activity (LcV: MLR 98 and 147, respectively) [35]. 

Moreover, using bioactivity-guided separation of the cytotoxic ethyl acetate-soluble fraction, lamellarin β (**90**) (Figure 10) and two known lamellarins G (**60**) and L (**61**) were isolated from Indian *Didemnum* sp. Lamellarin β (**90**) exhibited cytotoxic activity against human promyelocytic leukemia HL-60 with IC_50_ 10.1 μM in an MTT colorimetric assay [37]. Chemical analysis of *D. ternerratum*, collected from Tonga, resulted in the purification of six new lamellarin sulfates, lamellarin K-20-sulfate (**91**), lamellarin E-20-sulfate (**92**), lamellarin A3-20-sulfate (**93**), lamellarin B1-20-sulfate (**94**), lamellarin d-8-sulfate (**95**), and lamellarin B2-20-sulfate (**96**) (Figure 10 and Figure 11). In a 48 h MTS cell proliferation assay, compound **95** exhibited moderate cytotoxic activity against human colon carcinoma cell line HCT-116 with IC_50_ value of 9.7 μM, whereas all other compounds exhibited only weak activity [38].

**Figure 12:**
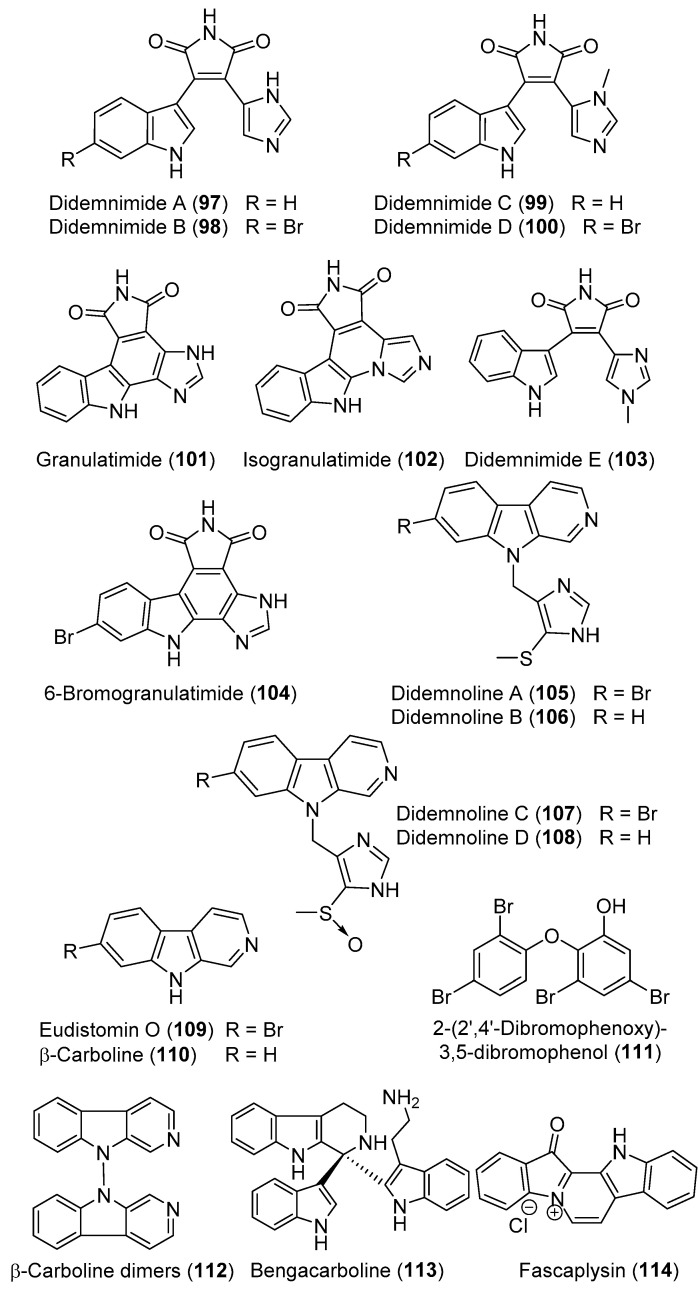
Chemical structures of compounds **97–114**.

Using an ecologically relevant assay as a guide for isolation of the feeding deterrent compounds from the active dichloromethane-methanol extract, four novel indole-maleimide-imidazole alkaloids, didemnimides A–D (**97**–**100**) (Figure 12), were isolated from *D. conchyliatum.* Didemnimide (**100**) was found to deter feeding of the carnivorous wrasse *Thalassoma bifasciatum* at natural concentrations in aquarium assays [39]. Additionally, using ELISA-based high-throughput bioassay for targeting G2 cell cycle checkpoint inhibitors, the active extract of the Brazilian *D. granulatum* was selected. A bioassay-guided fractionation of the extract resulted in the isolation of the alkaloids granulatimide (**101**) and isogranulatimide (**102**) together with didemnimides A (**97**), D (**99**), E (**103**), and 6-bromogranulatimide (**104**) (Figure 12). Compounds **101** and **102** displayed inhibitory activity for G2 cell cycle checkpoint and combined with a DNA damaging agent selectively kill p53-cancer cells [40,41]. 

The β-carboline alkaloids, didemnolines A–D (**105**–**108**) (Figure 12), were reported from an ascidian of the genus *Didemnum* collected from northern Maxima Islands along with eudistomin O (**109**), β-carboline (**110**), and 2-(2′,4′-dibromophenoxy)-3,5-dibromophenol (**111**) (Figure 12). Didemnolines A–C (**105**–**107)** exhibited moderate cytotoxic effects against human epidermoid carcinoma (KB) cells with IC_50_ values of 16.3, 10.2, and 0.72 μM, respectively. Further, didemnolines A and C (**105** and **107**) exhibited antimicrobial effects towards *Bacillus subtilis*, *Staphylococcus aureus*, *E. coli*, and two strains of *Saccharomyces cerevisiae* (RS188N and RS322Y) with inhibition zones ranging from 7 to 23 mm at 100 µg/6 mm disc [42]. A β-carboline dimer (**112**) (Figure 12) has been also isolated from an Australian *Didemnum* sp. [43]. From the cytotoxic (HCT 116) CHCl_3_ fraction of the methanolic extract of the Fijian *Didemnum* sp., a β-carboline derivative, bengacarboline (**113**) (Figure 12), was isolated from the along with fascaplysin (**114**) (Figure 12). Compound **113** displayed cytotoxic activity toward a 26 cell line human tumor panel with a mean IC_50_ of 2.69 μM and inhibited the catalytic activity of topoisomerase II at 32 μM [44]. 

**Figure 13:**
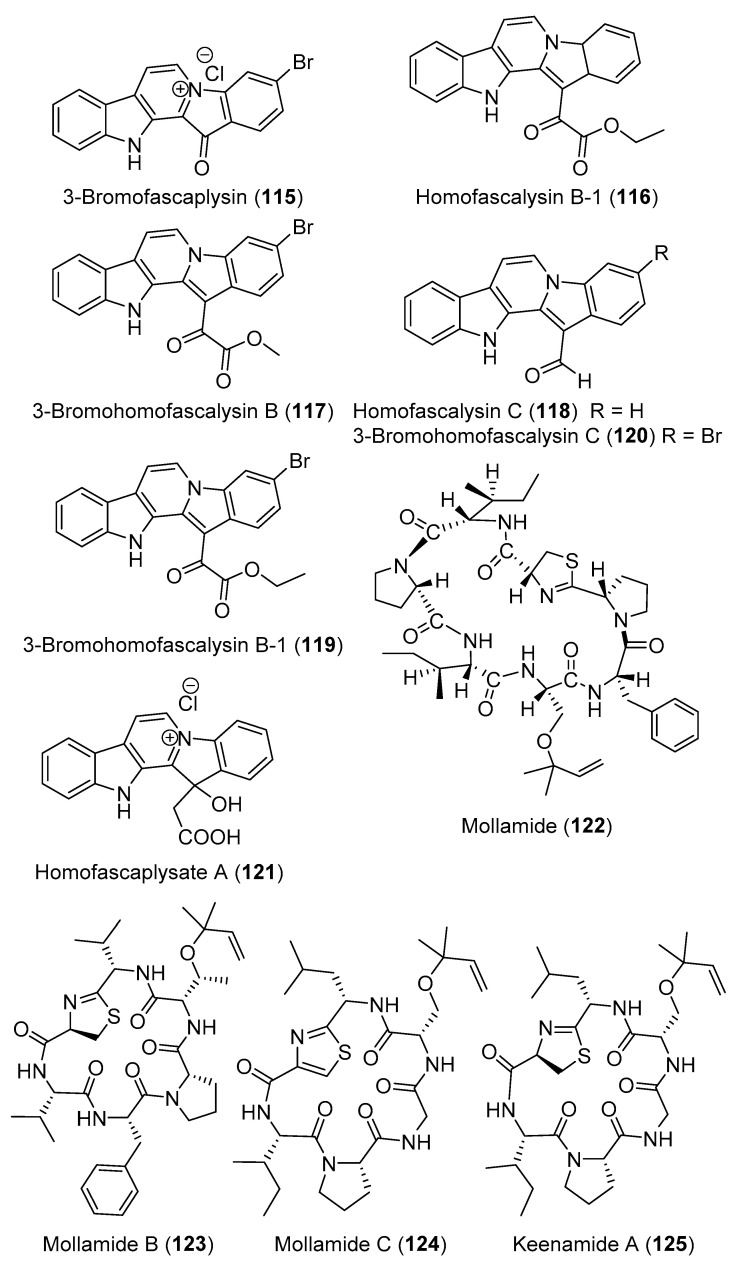
Chemical structures of compounds **115–125**.

Chemical investigation of two specimens of *Didemnum* sp. led to the identification of eight fascaplysin derivatives including fascaplysin (**114**) (Figure 12), 3-bromofascaplysin (**115**) (Figure 13), homofascaplysin B-1 (**116**), 3-bromohomofascaplysin B (**117**), homofascaplysin C (**118**), 3-bromohomofascaplysin B-1 (**119**), 3-bromohomofascaplysin C (**120**), and homofascaplysate A (**121**) (Figure 13). Using a disk diffusion soft agar colony formation assay, fascaplysin (**114**) was the most potent compound examined and exhibited selectivity towards murine tumor, while 3-bromo-fascaplysin (**115**) was not selective toward the tested cells [45].

**Figure 14:**
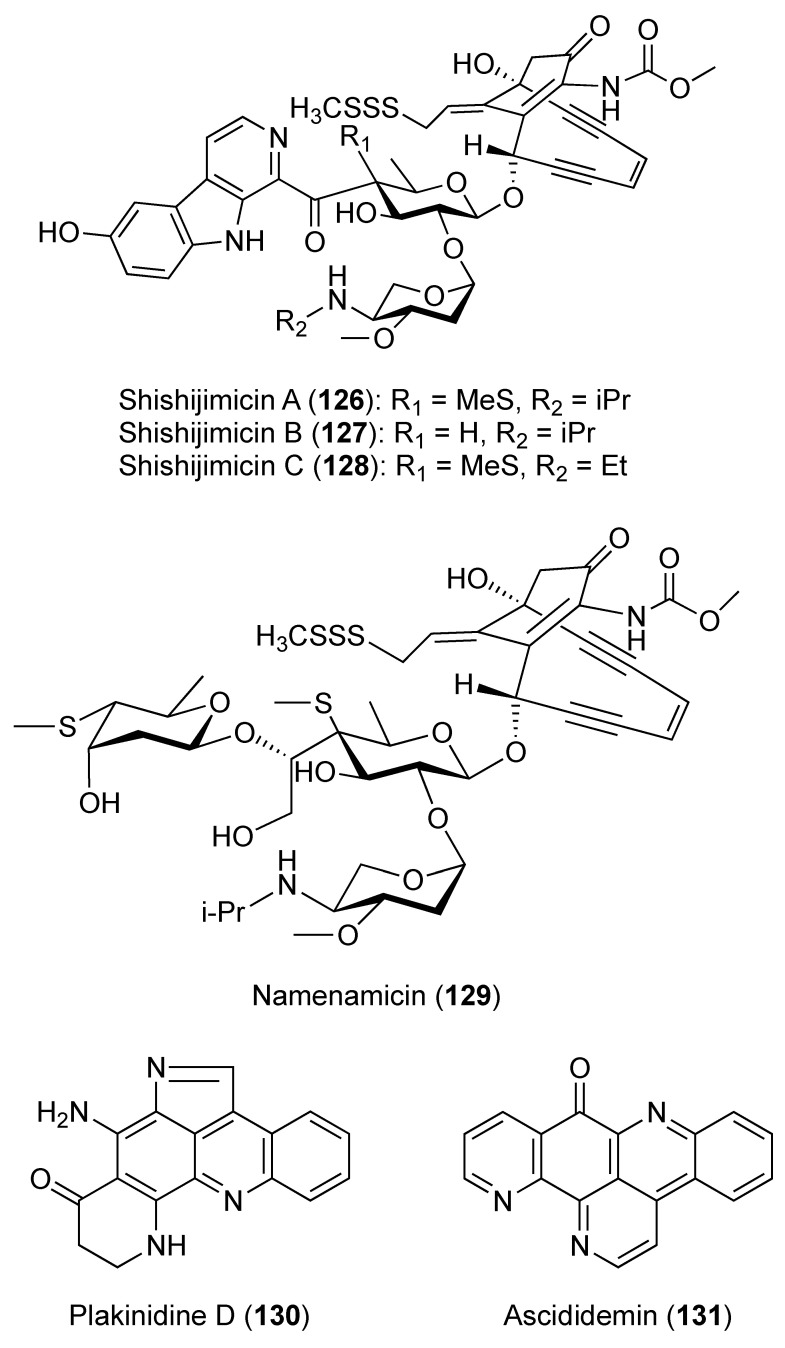
Chemical structures of compounds **126–131**.

A cyclic heptapeptide, mollamide (**122**) (Figure 13), was isolated from *D. molle* and displayed a cytotoxic effect with an IC_50_ of 1.2 μM toward P388 (murine leukemia) and 3.0 μM toward A549 (human lung carcinoma). Mollamide also inhibited the RNA synthesis with an IC_50_ of approximately at 1.23 μM [46]. Moreover, cyclic hexapeptides mollamides B and C (**123** and **124**) and keenamide A (**125**) (Figure 13) were reported from the Indonesian *D. molle*. Mollamide B (**123**) displayed significant growth inhibition of 29%, 44%, and 42% when tested at 100 μM against the non-small cell lung cancer cell line (H460), the breast cancer cell line (MCF7), and the CNS cancer cell line (SF-268). However, when evaluated by the National Cancer Institute (NCI) in the 60-cell-line panel, none of the tested cell lines displayed any sensitivity to mollamide B that exceeded the mean [47]. By contrast, when tested in an in vitro disk diffusion assay that aims to identify differential cell killing among nine cell lines (two leukemias, five solid tumors, a murine, and a human normal cell line), mollamide C (**124**) showed against L1210, human colon HCT-116, and human lung H125 a unit zone differential value of 100 and against murine colon 38 a value of 250. Therefore, **124** was not considered as solid-tumor selective [47]. Shishijimicins A–C (**126**–**128**) (Figure 14) and namenamicin (**129**) (Figure 14) were isolated from the Japanese *D. proliferum*. Compounds **126**–**129** exhibited strong cytotoxic effects toward various tumor cell lines including *Helacyton gartleri* (HeLa) (IC_50_ values are 0.000034, 0.0000019, 0.0000034, and 0.0000063 μM), fibroblast cell line 3Y1 (IC_50_ values are 0.000013, 0.000021, 0.0000032, and 0.0000048 μM), and P-388 mouse leukemia (IC_50_ values are 0.0000033, 0.0000005, 0.000002, and 0.0000017 μM) [48]. It was postulated that shishijimicins A–C (**126**–**128**) cleave DNA as in the case of other enediyne antibiotics including namenamicin (**129**) [48]. A pyrroloacridine alkaloid, plakinidine D (**130**) (Figure 14), was isolated from *D. rubeum*, along with 3,5-diiodo-4-methoxyphenethylamine (**27**) and ascididemin (**131**) (Figure 14). Compound **130** showed cytotoxicity to the human colon tumor cell line HCT-116 at 25 μM [49,50]. A unique pentacyclic aromatic alkaloid, ascididemin (**131**), isolated from Okinawan *Didemnum* sp., had strong antineoplastic activity toward L1210 murine leukemia cells with IC_50_ value of 1.37 µM. 

Ascididemin was also seven times more potent than caffeine (a known Ca releaser) in the Ca releasing activity in sarcoplasmic reticulum [51]. An iododinated phenethylamine derivative, 3,5-diiodo-4-methoxyphenethylamine (**27**) (Figure 7) was reported from an unidentified *Didemnum* sp., collected from the northwest end of Cocos Lagoon. Compound **27** showed antifungal effects versus *Candida albicans* and was slightly cytotoxic toward L1210 with IC_50_ of 49.7 µM [52]. 

**Figure 15:**
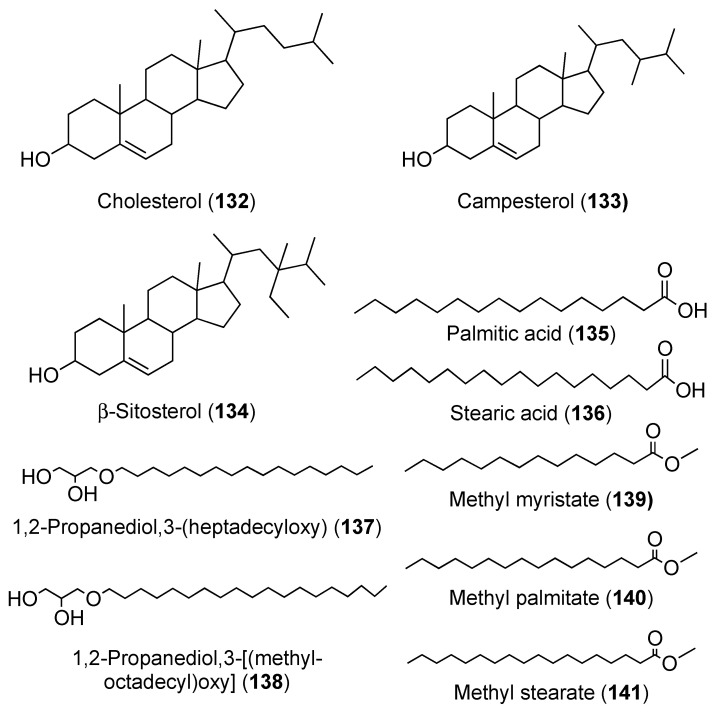
Chemical structures of compounds **132–141**.

Chemical analysis of *D. psammatodes* resulted in the isolation of 14 compounds including four steroids, stigmasterol (**18**) (Figure 6), cholesterol (**132**) (Figure 15), campesterol (**133**), and β-sitosterol (**134**); two fatty acids, palmitic acid (**135**) and stearic acid (**136**); two nucleosides, thymidine (**21**) and 2′-deoxyguanosine (**23**); three glyceryl ethers including 1,2-propanediol,3-(heptadecyloxy) (**137**), batyl alcohol (**19**), and 3-[(methyloctadecyl)oxy] (**138**) and a mixture of three methyl esters including methyl myristate (**139**), methyl palmitate (**140**), and methyl stearate (**141**) (Figure 15). Compounds **139** and **141** displayed cytotoxic effect towards T-cell leukemia (Molt-4 cells) with IC_50_ values of 12.9 and 15.9 μM. In addition, they were active against acute promyeloblastic leukemia (HL-60) with IC_50_ values of 13.0 and 11.4 μM, respectively. Compound **140** was cytotoxic to Molt-4 with an IC_50_ value of 9.4 μM [53].

**Figure 16:**
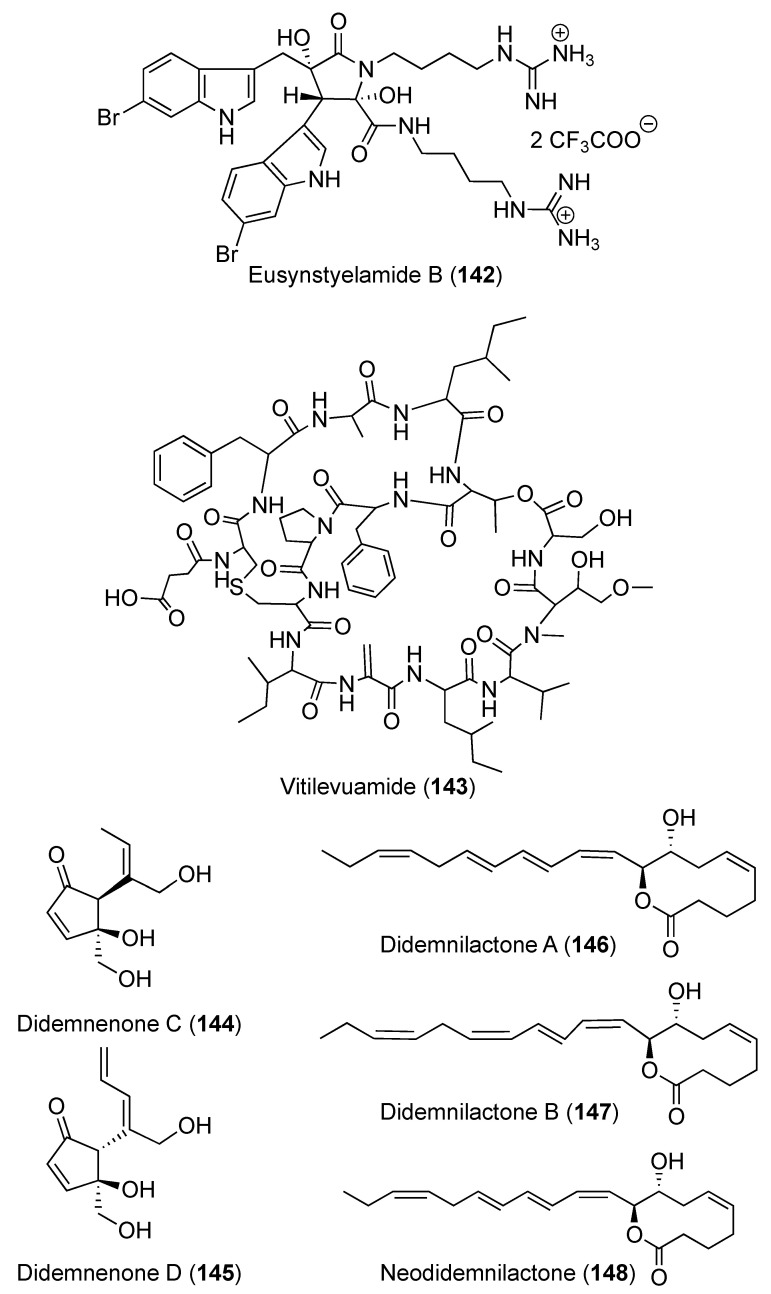
Chemical structures of compounds **142–148**.

Using a bioassay-guide fractionation of the active extract against breast cancer cells (MDA-MB-231), eusynstyelamide B (**142**) (Figure 16), a bis-indole alkaloid, was isolated from *D. candidum*. Compound **142** displayed antineoplastic activity against MDA-MB-231 cells with an IC_50_ value of 5.0 μM [54]. Further, the authors claim that **142** induced cell death of MDA-MB-231 cells through apoptosis [54]. A bicyclic depsipeptide, vitilevuamide (**143**) (Figure 16), was isolated from *D. cuculiferum.* Vitilevuamide (**143**) displayed powerful cytotoxic effects towards colon cancer cell line HCT 116 (IC_50_ 0.0062 μM); adenocarcinoma cell line A549 (IC_50_ 0.12 μM); malignant melanoma cell line SK-MEL-5 (IC_50_ 0.31 μM) and kidney carcinoma cell line A498 (IC_50_ 3.12 μM) [55]. Two cyclopentenone metabolites didemnenones C and D (**144** and **145**) (Figure 16) were isolated from *D. voeltzkowi*. Both compounds displayed in vitro cytotoxic activity against L1210 murine leukemia cell line with IC_50_ values of 28.2 and 26.6 μM, respectively [56]. Three fatty acid metabolites, didemnilactones A and B (**146** and **147**) along with neodidemnilactone (**148**) (Figure 16), were isolated from *D. moseleyi*. Didemnilactones A and B (**146** and **147**) presented moderate inhibitory activities against 5-lipoxygenase and 15-lipoxygenases of human polymorphonuclear leukocytes with IC_50_ of 9.4 and 8.5 μM, respectively, while **147** was weakly active against 15-lipoxygenases with IC_50_ of 41 μM. [57]. Didemnilactone A (**146**) and neodidemnilactone (**148**) had weak binding activity to leukotriene B4 receptors of human polymorphonuclear leukocyte membrane fractions with IC_50_ of 1.38 and 3.50 μM, respectively [57]. 

**Figure 17:**
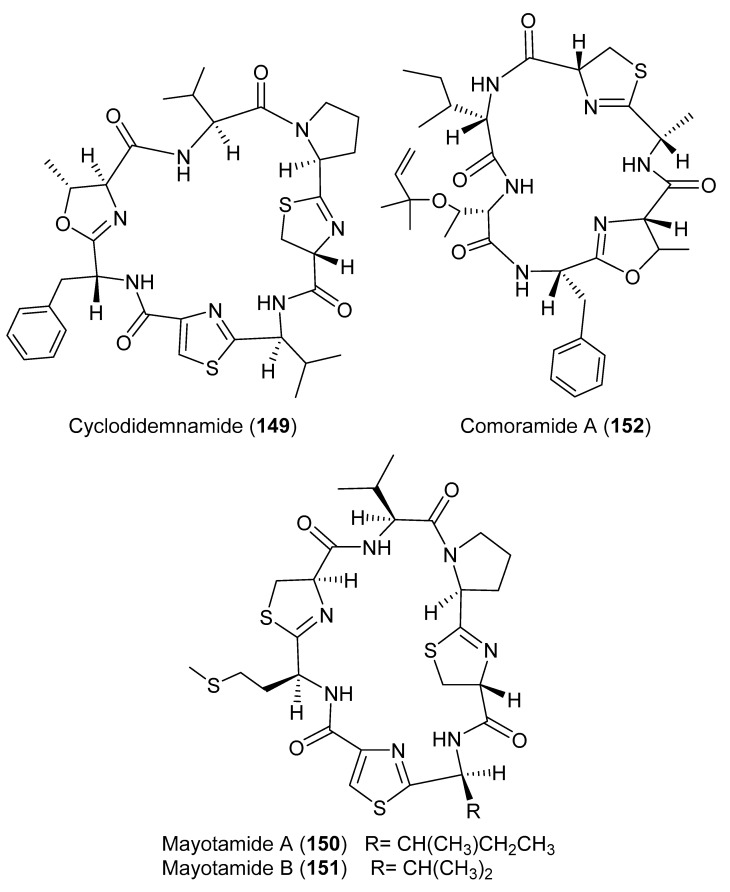
Chemical structures of compounds **149–152**.

**Figure 18:**
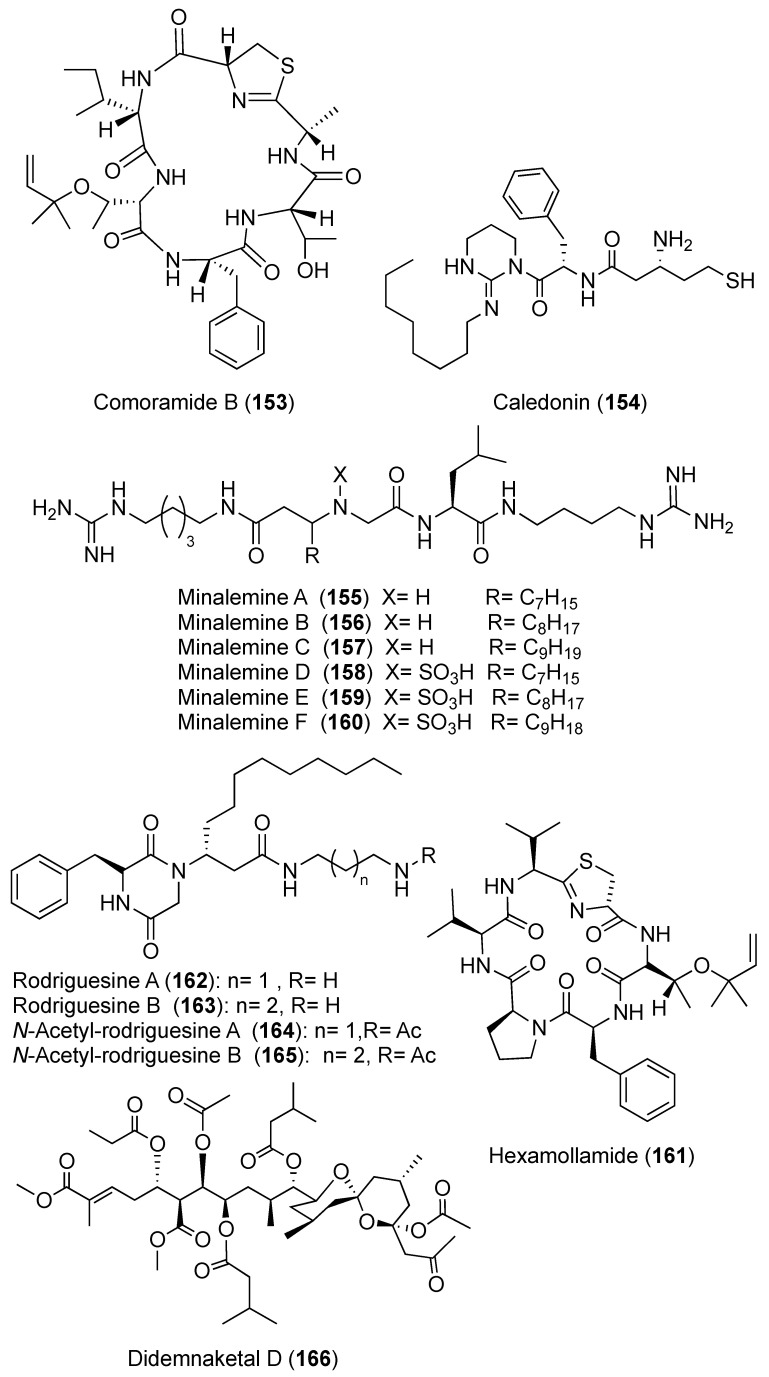
Chemical structures of compounds **153–166**.

During a chemical analysis of *D. molle* obtained from the Philippine Islands, a cyclic heptapeptide cyclodidemnamide (**149**) (Figure 17) was identified. Compound **149** displayed a weak cytotoxic activity against human colon tumor cells HCT-116 with ED_50_ of 23.0 μM [58]. Two cyclic heptapeptides, mayotamides A and B (**150** and **151**) (Figure 17) along with two cyclic hexapeptides comoramides A (**152**) (Figure 17) and B (**153**) (Figure 18), were isolated from two specimens of *D. molle* gathered at two locations in the lagoon of Mayotte. All compounds exhibited modest cytotoxic activity against many tumor cells (A549, HT29, and MEL-28) with IC_50_ ranging from 4.48 to 14.97 μM [59]. A unique modified peptide, caledonin (**154**) (Figure 18) was isolated from *D. rodriguesi* and displayed an in vitro cytotoxic activity effect against KB cells with 85% inhibition at 20.44 μM [60]. From the same *Didemnum* sp., six guanine metabolites, minalemines A–F (**155**–**160**) (Figure 18), were reported. Compounds **158** and **160** are sulfamic acid derivatives of **155** and **157** [61]. Moreover, a hexapeptide, hexamollamide (**161**) (Figure 18), was reported from an Okinawan *D. molle*, displaying a moderate cytotoxic activity against HeLa S3 cells with an IC_50_ value of 24.4 μM [62].

### 3.2. Compounds with Antimicrobial Activities (Table S2)

#### 3.2.1. Compounds with Antibacterial Activities (Table S2)

Four diketopiperazines alkaloids, rodriguesines A and B (**162** and **163**), *N*-acetylrodriguesine A (**164**) and *N*-acetylrodriguesine B (**165**) (Figure 18), were isolated from two tunicates of the genus *Didemnum* collected from Baía de Todos os Santos, Salvador, Bahia state, Brazil. A mixture of rodriguesines A and B (**162** and **163**) showed weak activity against different pathogenic microbial strains using broth microdilution assay (Table 1) [63]. Exceptionally, the mixture displayed strong activity against *Pseudomonas aeruginosa* P1 with MIC value of 4.3 μg/mL. Interestingly, the mixture was more active against the antibiotic-resistant strains than strains from standard collections (ATCC or NTCC) [63]. The authors did not use any antibiotic or antifungal standard(s) for comparison purposes in this experiment.

**Figure 19:**
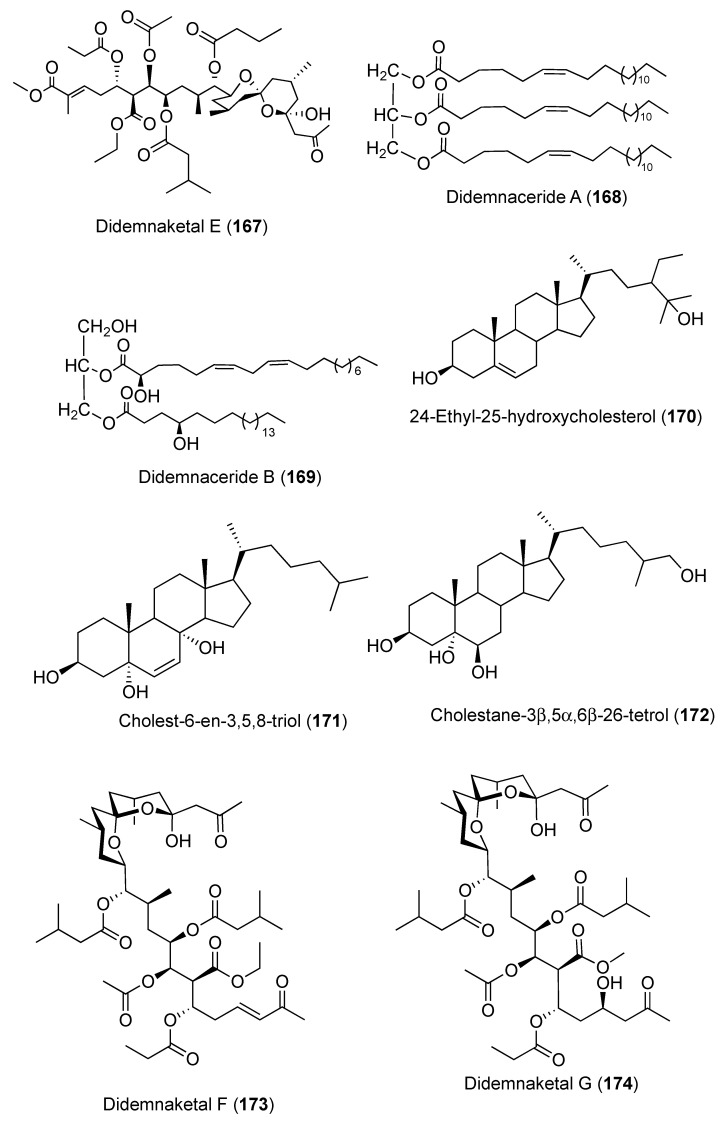
Chemical structures of compounds **167–174**.

**Figure 20:**
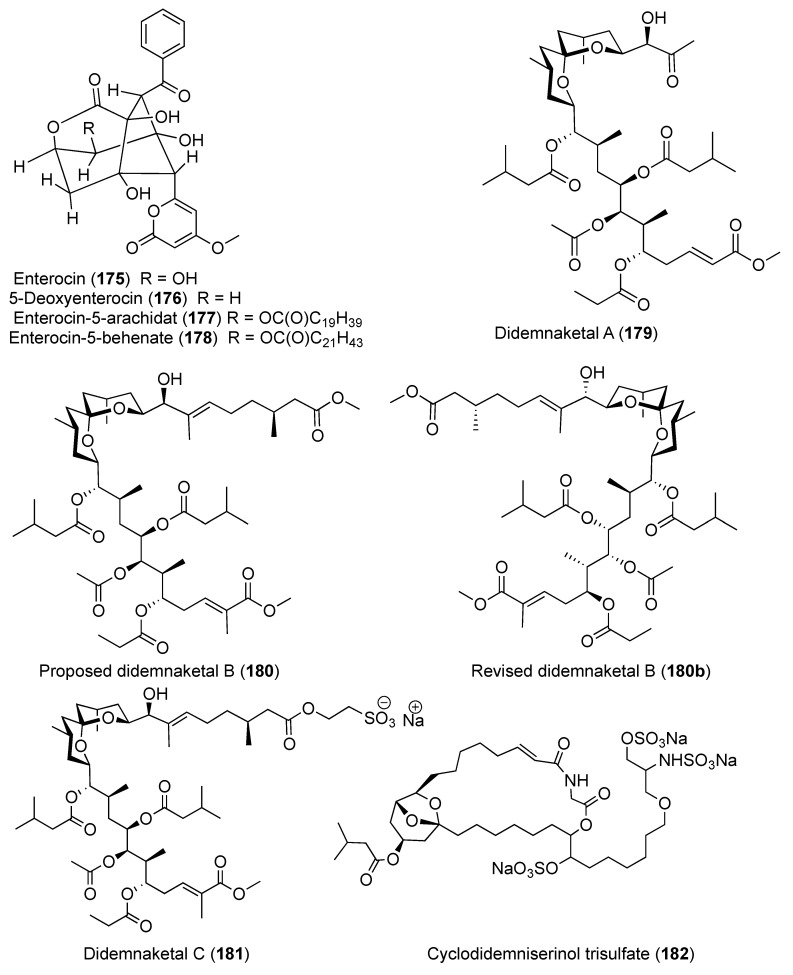
Chemical structures of compounds **175–182**.

Two spiroketals, didemnaketals D (**166**) (Figure 18) and E (**167**) (Figure 19), were isolated from a Red Sea *Didemnum* sp. (note that the stereochemistry of the didemnaketal series has been called into question; see below). In a disc diffusion assay at 20 µg/6-mm paper disc, Didemnaketals D (**166**) exhibited a modest antimicrobial activity with inhibition zone of 11 mm against *S. aureus* (ATCC 6538), while Didemnaketal E (**167**) showed a similar inhibition zone (11 mm) against *B. subtilis* (ATCC CC33) at the same concentration. Moreover, **166** and **167** showed a protein-kinase inhibitory activity towards the kinases CDK5, CK1, DyrK1A, and GSK3 with IC_50_ > 10.9 and 11.5 µM, respectively [64]. Additional analysis from the same species afforded two glycerides, didemnacerides A and B (**168** and **169**) (Figure 19), along with three sterols including 24-ethyl-25-hydroxycholesterol (**170**), cholest-6-en-3,5,8-triol (**171**), and cholestane-3β,5α,6β-26-tetrol (**172**) (Figure 19) [65]. Furthermore, two additional spiroketals, didemnaketals F and G (**173** and **174**) (Figure 19) were reported from the Red Sea *Didemnum* sp. Didemnaketal F (**173**) exhibited strong anti-microbial activity toward *E. coli* and *C. albicans* with inhibition zones of 20 and 24 mm at a concentration of 100 μg/disc in a disc diffusion assay, whereas didemnaketal G (**174**) exhibited modest activity towards *E. coli* and *C. albicans* with inhibition zones of 7 and 17 mm at the same concentration. Further, both compounds showed moderate cytotoxic activity against HeLa cells with IC_50s_ of 49.9 and 14.0 μM, respectively [66]. Four metabolites, derivatives of the bacterial antibiotic enterocin, were reported from a Western Australian *Didemnum* sp. including enterocin (**175**), 5-deoxyenterocin (**176**), enterocin-5-arachidate (**177**), and enterocin-5-behenate (**178**) (Figure 20) [67]. 

#### 3.2.2. Compounds with Antiviral Activities (Table S2)

Two anti-HIV spiroketals were reported from *Didemnum* sp. obtained from Palau, didemnaketals A and B (**179** and **180**) (Figure 20). Both compounds might be artifacts resulting from the methanolysis of didemnaketal C (**181**) (Figure 20) after prolonged storage (11 years) of the tunicate specimen in methanol. Compounds **179** and **180** exhibited strong HIV-1 protease inhibitory effect with IC_50_ values of 2 and 10 μM, respectively [68,69]. Recently, the configuration of the whole didemnaketal series has been called into question by the total synthesis of didemnaketal B (**180**), revising the configuration of didemnaketal B to (**180b**) (Figure 20) [70]. Since the configurations of most didemnaketals are based upon structure **180**, they should likely also be revised. 

**Figure 21:**
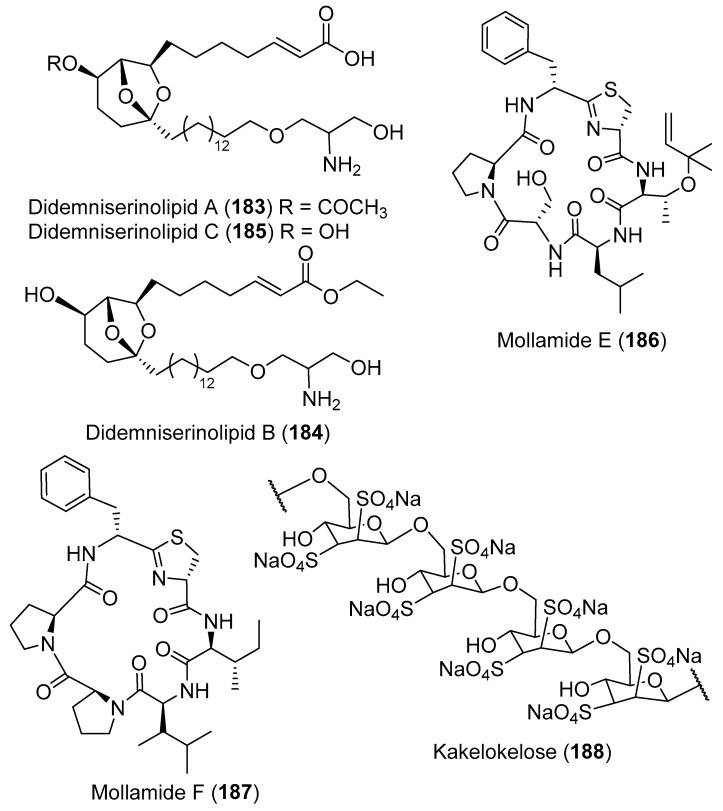
Chemical structures of compounds **183–188**.

The chemical investigation of the *D. guttatum* resulted in the isolation of cyclodidemniserinol trisulfate (**182**) (Figure 20). This compound is closely related to didemniserinolipid A (**183**) (Figure 21) which is obtained from an Indonesian *Didemnum* sp. [71]. There are some notable variations between the structures of **182** and **183** with the presence of an additional ring containing a glycine unit and the presence of sulfate groups in **182** [72]. Furthermore, didemniserinolipids B and C (**184** and **185**) (Figure 21) are reported from the same tunicate species [71]. Tracing the active fraction in an HIV integrase assay through a bioassay-guided purification of the methanolic extract of *Didemnum* sp. led to the isolation of didemniserinolipid A (**183**). It displayed inhibitory effects against HIV-1 protease and MCV topoisomerase with an IC_50_ of 100.5 and 120.6 μM, respectively [72]. A bioassay-guided fractionation of the extract of *D. molle* resulted in the isolation of two thiazoline peptides, mollamides E and F (**186** and **187**) (Figure 21), and the tris-phenethyl urea, molleurea A (**5**) (Figure 4) [72]. Compound **187** displayed a modest anti-HIV activity in both HIV integrase inhibition assay and a cytoprotective cell-based assay with IC_50_ values of 39 and 78 μM, respectively, while compound **5** was active only in the cytoprotective cell-based assay with IC_50_ of 60 μM [73]. A unique sulfated mannose homopolysaccharide, kakelokelose (**188**) (Figure 21), was reported during an investigation of mucous secretion of the Pacific *D. molle*. Compound **188** displayed a remarkable anti-HIV action determined 100% potential to inhibit infection with CEM cells by HIV strain RF at 0.20 μM, while no cytotoxicity against CEM cells at a concentration of 10.25 μM was observed [74]. 

**Figure 22:**
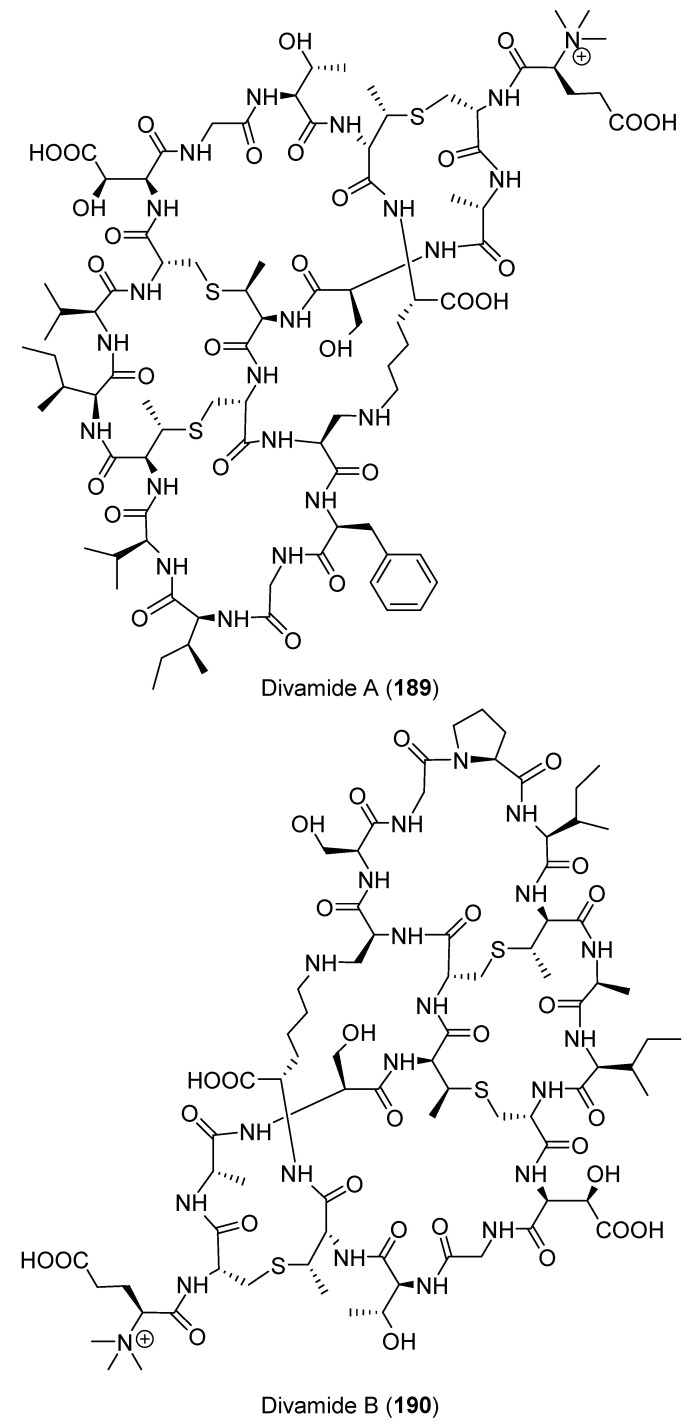
Chemical structures of compounds **189** and **190**.

Using a bioassay-guided fractionation of the anti-HIV extract of *D. molle* collected in the Eastern Fields of Papua New Guinea, two anti-HIV compounds, divamides A (**189** and **190**) (Figure 22) were isolated [75]. Insufficient material was obtained for full structure elucidation, so metagenome sequencing revealed a biosynthetic pathway encoded in symbiotic *Prochloron* bacteria. The pathway was expressed in *E. coli*, leading to material for full structure elucidation and pharmacological testing. Compound **189** had an IC_50_ of 0.225 μM against HIV, with a cytotoxic CC_50_ of 2.64 μM against CEM-TART cells. Compound **190** was essentially inactive in both assays at concentrations < 10 μM [75].

#### 3.2.3. Compounds with Antifungal Activities (Table S2)

During the chemical investigation of methanolic extract of Australian *Didemnum* sp., (*R*)-(*E*)-1-aminotridec-5-en-2-ol (**191**) was isolated, displaying modest activity toward *Candida albicans* (9-mm zone of inhibition at 50 μg/disk) [76] In addition, two minor compounds were characterized as their *N*-Boc derivatives, l-(*N*-Boc-amino)tridec-4-en-2-ol (**192**) and l-(*N*-Boc-amino)tridec-5-en-2-ol (**193**) [76].

### 3.3. Compounds with Antimalarial and Antitrypanosomal Activities (Table S3)

**Figure 23:**
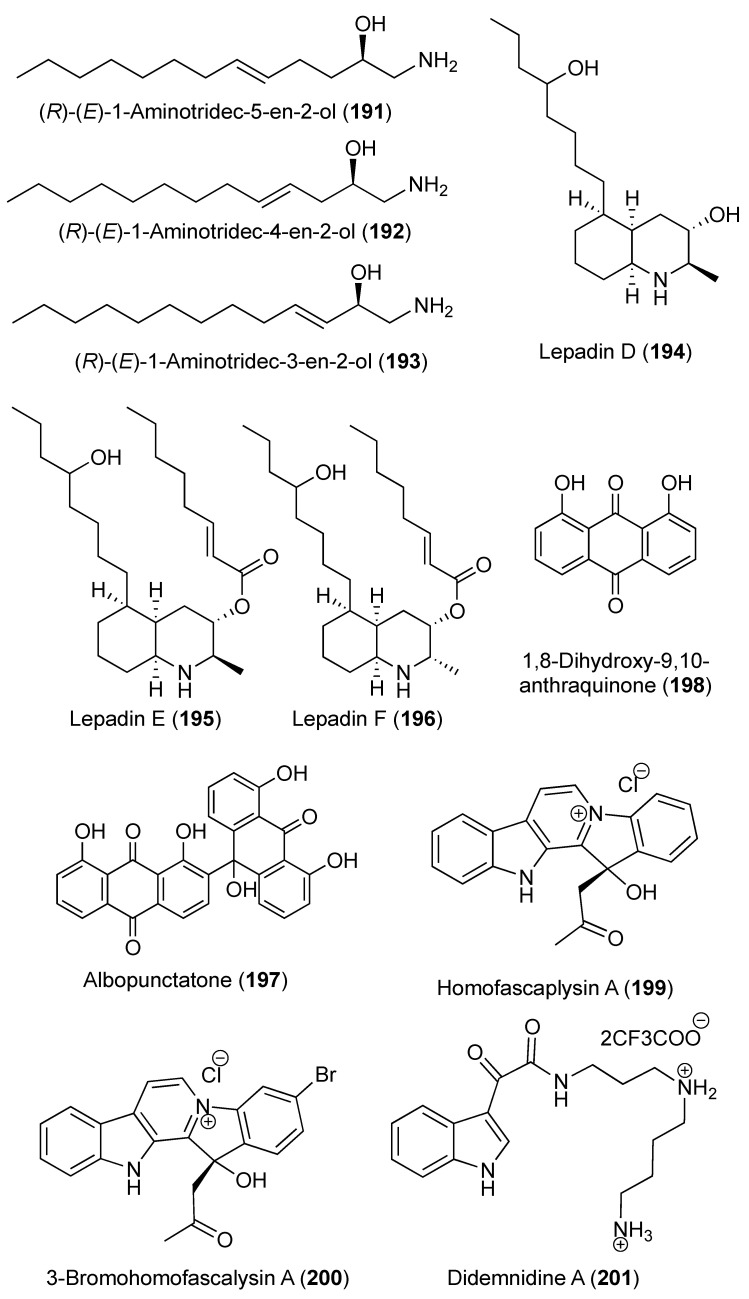
Chemical structures of compounds **191–201**.

Three decahydroquinolines metabolites were reported from tropical marine *Didemnum* sp., lepadins D–F (**194**–**196**) (Figure 23). All compounds displayed antimalarial effect against human malaria parasite *Plasmodium falciparum* with an IC_50_ of 20.5, 0.95, and 0.47 μM against chloroquine-resistant strain K1 and with IC_50_ values of 50.7, 2.13, and 0.71 μM against chloroquine-sensitive strain NF54. The compounds displayed antitrypanosomal activities with IC_50_ values of 125.2, 5.2, and 6.17 μM against *Trypanosoma cruzi*, and IC_50_ values of 18.8, 0.9, and 0.54 μM for *Trypanosoma rhodesiense* [77]. The anthrone-anthraquinone, albopunctatone (**197**), and 1,8-dihydroxy-9,10-anthraquinone (**198**) (Figure 23), were isolated from an Australian *D. albopunctatum*. Compound **197** exhibited moderate antiplasmodial effect toward chloroquine-resistant and sensitive strains of *P. falciparum* in a Malaria Imaging Assay with IC_50_ of 5.3 and 4.4 μM, respectively [78]. On the other hand, **198** was inactive against both strains at dose up to 40 μM. Further, both **197** and **198** were inactive against cancer and normal cell lines and the kinetoplastid *Trypanosoma brucei brucei*, suggestive the selectivity of **197** against *P. falciparum* [77].

**Figure 24:**
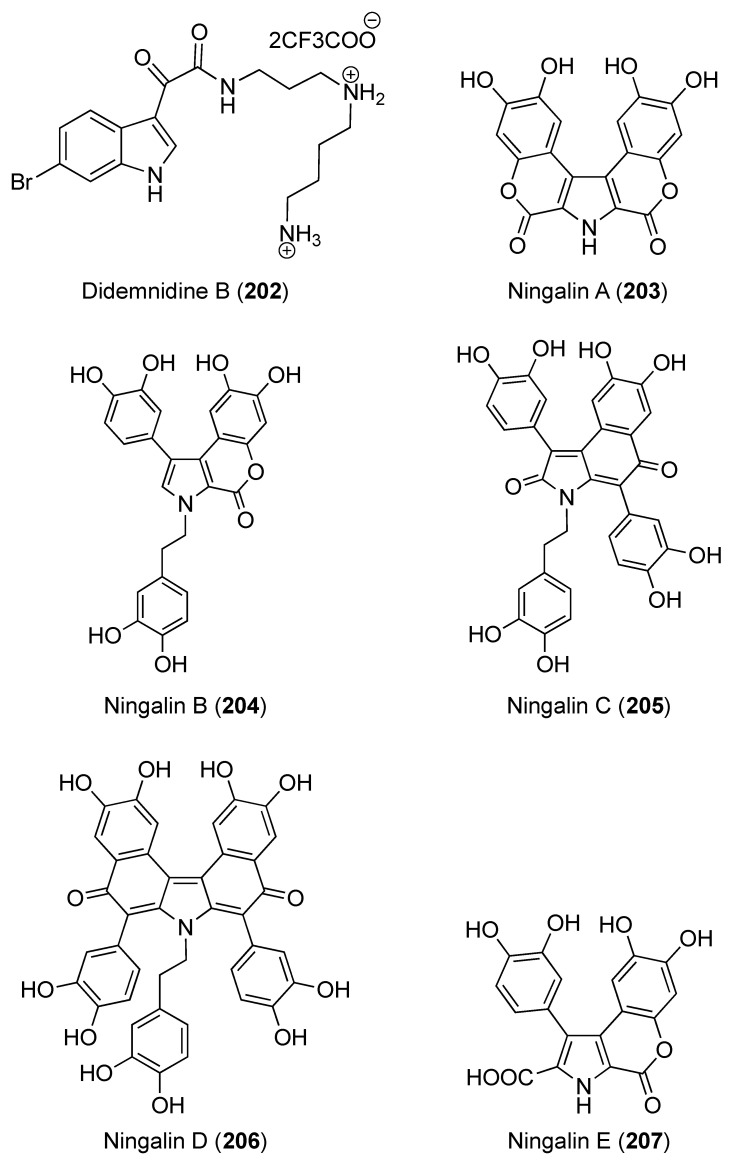
Chemical structure of compound **202–207**.

The fascaplysin alkaloid analogues homofascaplysin A (**199**), 3-bromohomofascaplysin A (**200**) (Figure 23), and fascaplysin (**114**) were isolated from a Fijian *Didemnum* sp. Using a flow cytometric analysis of malaria parasite growth, the antiplasmodial activity of homofascaplysin A (**199**) was evaluated. Compound **199** displayed an IC_50_ of 0.55 ± 0.11 nM versus ring-stage parasites and 105 ± 38 nM versus all live parasites. Therefore, **199** represents a potential agent against drug-resistant malaria [79]. Mollamide B (**123**) showed a moderate anti-malarial activity versus *P. falciparum* (D6 clone and W2 clone), with IC_50_ values of 0.28 and 3.0 μM, respectively. Compound **123** also exhibited minor effects versus *Leishmania donovani* with IC_50_ and IC_90_ values of 25.8 and 50.2 μM, respectively, and against HIV-1 in human PBM cells with an EC_50_ value of 48.7 μM in vitro [47]. Two indole spermidine alkaloids, didemnidines A (**201**) (Figure 23) and B (**202**) (Figure 24), were isolated from New Zealand *Didemnum* sp. Using a whole organism parasite assay, compound **201** showed moderate in vitro growth inhibitory effect against *P. falciparum* with IC_50_ of 15 μM. In addition, **201** showed moderate cytotoxicity against the nonmalignant L6 cell line, indicating a limited selectivity toward *P. falciparum* [80].

### 3.4. Compounds with Antidiabetic Activity (Table S4)

A phenylalanine derivative, *N,N*′-diphenethylurea (**11**) (Figure 5), was isolated from an Okinawan *D. molle*. Compound **11** enhanced adipocyte differentiation and PPARγ activity as a weak ligand and an insulin signal, perhaps via the phosphoinositide-3-kinase/Akt signal pathway, in 3T3-L1 cells, which was speculated to be of potential use in treating diabetes [81]. 

### 3.5. Compounds that Affect the Central Nervous System (Table S5)

**Figure 25:**
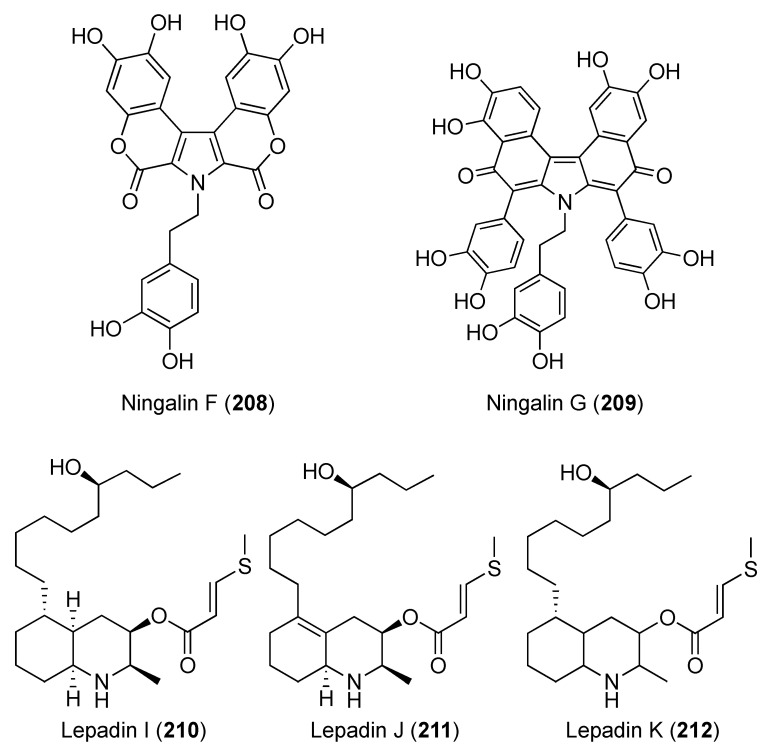
Chemical structures of compounds **208–212**.

The aromatic alkaloids derivatives, ningalins A–G (**203**–**209**) (Figure 24 and Figure 25) were reported from a Western Australian *Didemnum* sp. together with pyrrole alkaloids lamellarins A6 (**85**), G (**60**), and Z (**62**) (Figure 10) [82,83]. These compounds were evaluated for their kinase inhibitory effects versus the neurodegenerative disease. The results showed that ningalins B (**204**), E (**207**), and F (**208**) possessed modest inhibitory effects against CK1δ and GSK3β with IC_50_ of 0.8–3.9 μM, while CDK5 was only inhibited by ningalins B (**204**) with IC_50_ of 2.6 μM [82]. Furthermore, lamellarins G (**60**) and A6 (**85**) showed inhibition of CDK5 with IC_50_ of 5.6 and 1.0 μM, while lamellarins Z (**62**) inhibited CDK5 and GSK3β and with IC_50_ of 1.1 and 3.0 μM, respectively [83]. 

To understand the molecular interactions important for kinase inhibition by ningalins and lamellarins, docking studies were performed using the X-ray crystallographic structure of CDK5^D144N^/p25 in complex with aloisine. It was predicted that the ningalins preferentially bind in the ATP binding site, which is consistent with their broad inhibitory effects across the three kinases. Lamellarins, on the other hand, are predicted to prefer to bind in the ATP binding pocket making the lamellarins’ activity due to a nonspecific interaction with the kinases [83].

Three decahydroquinolines alkaloid derivatives lepadins I, J, and K (**210**–**212**) (Figure 25) were isolated from a Bahamian *Didemnum* sp. The compounds were evaluated in an anti-cholinesterase reporter assay using a modification of Ellman’s photometric method with physostigmine as a positive control. Lepadin I (**210**) exhibited butyrylcholinesterase (BuChE) inhibitory activity with an IC_50_ value of 3.1 μM versus physostigmine (IC_50_ 1.48 μM). Lepadin I also showed a minor acetylcholinesterase (AChE) inhibitory effect (10% at 100 μM), suggesting a non-specific activity [84]. 

## 4. Discussion: The Chemistry and Chemical Potential of *Didemnum*

**Figure 26:**
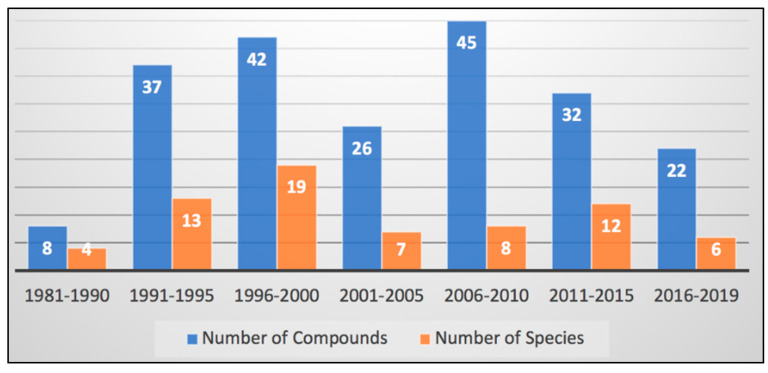
Number of compounds and species reported in the genus *Didemnum* literature between 1981 and 2019.

Since the first report of *Didemnum* secondary metabolites by Ireland, Durso, and Scheuer in 1981 [16], the genus has contributed at least 212 compounds. The field was most active during the period of 1993–2009, when 143 new compounds were reported (68%) from at least 45 species (65%) (Figure 26). However, new discoveries continue apace even through 2019, with 54 compounds (25%) from at least 18 species (26%). These species have been collected from locations around the world, focused on tropical regions (Figure 26). There is a notable scarcity of reports from the western coasts of all continents. These data indicate that genus *Didemnum* continues to be a rich source of secondary metabolites that are new to science and suggest potential locations for further discovery.

*Didemnum* has been an exceptional source of compounds with important therapeutic promise, with most interest so far involving potential anticancer compounds. Among these, the lamellarins have been the most extensively studied for their anticancer potential [85]. These compounds were initially discovered in a mollusk, *Lamellaria* sp., which likely concentrates the compounds from a tunicate diet [86]. The enediynes namenamicin and shishijimicins are exceptionally potent (picomolar) [48]. Structurally related enediynes have been FDA approved as their antibody-drug conjugates [87]. The structurally unusual, cyclic peptide vitilevuamide exhibited powerful, subnanomolar potency against a variety of cancer cell lines [55]. Another cyclic peptide, divamide A, inhibited HIV replication at ≈200 nM in cell lines [75]. 

There are many different chemical families found in *Didemnum* spp., but as in other tunicates a large percentage appear to be amino acid-derived. Virtually nothing is known about the biosynthetic origin of most of these compounds, with the exception of two classes of ribosomally synthesized and posttranslationally modified peptides (RiPPs) [88], which in both cases are produced by obligately symbiotic bacteria, *Prochloron* spp. [89]. One group of these belongs to a large family of mostly N-C circular peptides, the cyanobactins [90]. *Didemnum* spp. cyanobactins include didmolamides, anatollamides, minimide, mollamides, keenamide, comoramides, mayotamides, and hexmollamide, or a total of 16 compounds unique to genus *Didemnum* spp. Another class of *Didemnum* spp. RiPPs is a group of lanthipeptides, the divamides [75]. In both the cyanobactin and the divamide cases, the biosynthetic pathways were identified by metagenome sequencing. Bioinformatics methods localized the biosynthetic pathways to symbiotic *Prochloron* bacteria, and not to the host itself. The pathways were reconstructed using chemical synthesis of DNA, and the resulting plasmids were expressed in *Escherichia coli* in the laboratory, leading to lab-based synthesis of the natural products. This represents strong evidence that the symbiotic bacteria, and not the host, are responsible for making bioactive compounds isolated from the whole animals.

Beyond the RiPPs made by symbiotic *Prochloron* bacteria, several other *Didemnum* metabolites likely have a symbiotic origin, but no biosynthetic studies have yet been reported [87]. For example, enterocins and enediynes are very similar to, or even identical to, products isolated from cultivated bacteria [91,92]. These, and also compounds such as didemnaketals, appear to be of polyketide origin, of a class normally associated with bacterial metabolism. Vitilevuamide has several features that are hallmarks of both RiPP and nonribosomal peptide biosynthesis in bacteria. For most other *Didemnum* spp. compounds, the biosynthetic origin is not clear. In many of those cases, the hosts themselves may likely synthesize many of the compounds, rather than symbiotic bacteria. Biosynthesis of secondary metabolites by animal biochemistry is a barely explored, blooming field [93]. In those cases, in contrast to finding pathways from symbiotic bacteria, the tool required is transcriptomics, so that genes expressed by the animal are analyzed and subsequently their products are biochemically characterized [94].

Overall, the chemistry of *Didemnum* spp. is distinct, with several classes of compounds that have not yet been found in other organisms. However, many compounds bear striking similarities to those from other didemnid ascidians. For example, *Prochloron* spp. symbionts are widespread in Didemnidae, where they produce many cyanobactins [90]. Interestingly, vitilevuamide and enediynes have been found in at least two different genera of colonial tunicates, implicating potentially as-yet unidentified symbiotic bacteria that might be widespread within family Didemnidae [55,87].

Given the interest in compounds from *Didemnum*, it could be asked why there are not even more studies reported for this widespread genus. A major challenge stems from the biology of the organisms. While some *Didemnum* spp. grow as massive sheets that can coat shallow substrates in the sea, most animals consist of very small colonies (Figure 2). In the colonial didemnid ascidians, chemistry can vary between seemingly identical colonies collected in the same location [95]. Such variation can arise from the cryptic biodiversity prevalent in didemnid tunicates: most importantly, tunicates can be quite different species even though their appearances are identical. This has slowed the development of the field.

The methods of metagenome sequencing/transcriptomics and synthetic biology offer one potential avenue to access the diverse chemistry found in these abundant, yet tiny and variable, animal colonies. So far, in two cases involving RiPP biosynthesis within tunicates, painstaking efforts have led to the production of three *Didemnum* genus compounds—which are actually produced by symbiotic bacteria—in *E. coli* [23,75]. Developments in biotechnology may help to further access the potent, pharmaceutically promising agents from this ubiquitous genus. One key factor that has been found in studies of didemnid tunicates (although not focused on *Didemnum* spp.) was that the host taxonomy is most predictive of chemistry [14,96]. When chemical variation is observed in two identical looking didemnid tunicates, it is most likely that they are actually different species. This holds true even when the chemistry is made by bacteria, and not by the host animal. Thus, a better understanding of biology and ecology is a crucial ingredient in the discovery of new potential drugs from tunicates.

In summary, *Didemnum* spp. tunicates have been exceptional sources of biosynthetic and biochemical novelty applied to drug discovery. Even facing significant headwinds, new discoveries from *Didemnum* spp. and other tunicates from family Didemnidae continue apace.

## Figures and Tables

**Table 1:** Reported MIC values of compounds **162** and **163** against different pathogens [63].

Pathogenic Microbial Strain	MIC in µg/mL
*Staphylococcus aureus* ATCC 6538	62.5
*Staphylococcus aureus* ATCC 259223	22.6
ORSA 8	45.3
ORSA 108	91.0
*Escherichia coli* ATCCNTCC 861	125.0
*Escherichia coli* ATCC 259222	45.6
*Pseudomonas aeruginosa* ATCC 27853	22.6
*Pseudomonas aeruginosa* 13	45.3
*Pseudomonas aeruginosa* P1	4.3
*Candida albicans* ATCC 10231	n.a.
*Candida albicans* ATCC 36801 (serum type A)	125.0
*Enterococcus faecalis* ATCC 14506	125.0
*Streptococcus sanguinis* ATCC 15300	125.0
*Streptococcus sobrinus* ATCC 27607	125.0
*Streptococcus mutans* UA 159	62.5
*Streptococcus mutans* (clinical isolate 2.M7/4)	31.2

ORSA: Oxacillin-resistant *Staphylococcus aureus*; n.a.: not assigned.

## Supplementary Materials

The following are available online at https://www.mdpi.com/1660-3397/18/6/307/s1, Table S1: Compounds with antitumor/anticancer and antiproliferative activities, Table S2: Compounds with antimicrobial activities, Table S3: Compounds with antiprotozoal activities, Table S4: Compounds with antidiabetic activities, Table S5: Compounds that affect the central nervous system, Table S6: Compounds without any reported biological activities.
