# Supplementary material for: Secondary Metabolites of the Genus Didemnum: A Comprehensive Review of Chemical Diversity and Pharmacological Properties
Source: Mar Drugs. 2020 Jun 11;18(6):307. doi: 10.3390/md18060307 (PMC7344992; doi:10.3390/md18060307)
Supplement: Supplementary file 1 [file marinedrugs-18-00307-s001.pdf]

Supporting Information for

Secondary Metabolites of the Genus *Didemnum*: A Comprehensive Review of Chemical Diversity and Pharmacological Properties

Diaa T.A. Youssef [1,2,*], Hadeel Almagthali [1,3], Lamiaa A. Shaala[4,5,6] and Eric W. Schmidt [7,*]

1 Department of Natural Products, Faculty of Pharmacy, King Abdulaziz University, Jeddah 21589, Kingdom of Saudi Arabia

2 Department of Pharmacognosy, Faculty of Pharmacy, Suez Canal University, Ismailia 41522, Egypt

3 Department of Pharmacognosy, College of Pharmacy, Taif University, Al-Haweiah 21974, Kingdom of Saudi Arabia

4 Department Natural Products Unit, King Fahd Medical Research Center, King Abdulaziz University, Jeddah 21589, Kingdom of Saudi Arabia

5 Department of Medical Laboratory Technology, Faculty of Applied Medical Sciences, King Abdulaziz University, Jeddah 21589, Kingdom of Saudi Arabia

6 Suez Canal University Hospital, Suez Canal University, Ismailia 41522, Egypt

7 Department of Medicinal Chemistry, University of Utah, Salt Lake City, Utah 84112, USA

***** Correspondence: dyoussef@kau.edu.sa (DTAY); ews1@utah.edu (EWS); Tel.: (+966-548-535-344) (DTAY); (+1-801-585-5234) (EWS).

NOTE: In all below tables, yellow highlight indicates that the method used bioassay-guided fractionation.

Table S1. Compounds with antitumor/anticancer and antiproliferative activities**.**

Compound Name	Chemical Class	Source organism/Location	Solvent of Extraction	Purification of the compounds	Structural Determination	Ref.
**Lamellarin-ζ (69)**	Alkaloid	*Didemnum obscurum* / Tiruchandur coast in the Gulf of Mannar, Tamilnadu, India	MeOH	-TLC, CC (silica gel), GFC-Sephadex LH-20, C18 HPLC	-UV, IR, MS, and 1D NMR (^{1}H, ^{13}C) 2D NMR (HMQC, HMBC, NOESY)	33
**Lamellarin-η (70)**	Alkaloid	*Didemnum obscurum* / Tiruchandur coast in the Gulf of Mannar, Tamilnadu, India	MeOH	-TLC, CC (silica gel), GFC-Sephadex LH-20, C18 HPLC	-UV and IR, MS, 1D NMR (^{1}H, ^{13}C) and 2D NMR (HMQC, HMBC, NOESY)	33
**Lamellarin-φ (71)**	Alkaloid	*Didemnum obscurum* / Tiruchandur coast in the Gulf of Mannar, Tamilnadu, India	MeOH	-TLC, CC (silica gel), GFC-Sephadex LH-20, C18 HPLC	-UV and IR, MS, 1D (^{1}H, ^{13}C) and 2D NMR (HMQC, HMBC, NOESY)	33
**Lamellarin-χ (72)**	Alkaloid	*Didemnum obscurum* / Tiruchandur coast in the Gulf of Mannar, Tamilnadu, India	MeOH	-TLC, CC (silica gel), GFC-Sephadex LH-20, C18 HPLC	-UV and IR spectra, MS, 1D NMR (^{1}H and ^{13}C) and 2D NMR (HMQC, HMBC, NOESY)	33
		Didemnum sp. / Wasp Island, New South Wales	Aqueous EtOH	-LLPC and HPLC-UV-DAD	-UV, HRMS, 1D (^{1}H, ^{13}C) and 2D NMR (HMQC, HMBC, NOESY)	34
**Lamellarin K (74)**	Alkaloid	*Didemnum* sp. / Wasp Island, New South Wales	Aqueous EtOH	-LLPC, HPLC-UV-DAD	-UV, HRMS, 1D (^{1}H and ^{13}C) and 2D NMR (HMQC, HMBC, NOESY)	34
		Dark purple encrusting colonies of *Didemnum* sp. / South West Cay, Lihou Reef, off the North Queensland coast	MeOH/CH_2Cl_2	-VLC (silica gel) and RP HPLC	-HREIMS, 1D (1H, ^{13}C, INEPT and NOE) and 2D NMR (XHCORRD, HMQC, COLOC)	35
		Didemnum obscurum / Tiruchandur coast in the Gulf of Mannar, Tamilnadu, India	MeOH	-TLC, CC (silica gel), GFC-Sephadex LH-20, C18 HPLC	-UV, IR, MS, 1D (^{1}H, ^{13}C) and 2D NMR (HMQC, HMBC, NOESY)	33
		Didemnum sp. */* Australia	EtOH	-LLP, GPC-Sephadex LH-20, C18-Sep Pak, RP HPLC	-UV, HRMS, 1D (^{1}H, ^{13}C) and 2NMR (HETCOR, COLOC, COSY)	36
**Lamellarin J (76)**	Alkaloid	*Didemnum obscurum* / Tiruchandur coast in the Gulf of Mannar, Tamilnadu, India	MeOH	-TLC, CC (silica gel) (GFC-Sephadex LH-20), C18 HPLC	UV, IR, MS, 1D (^{1}H, ^{13}C) and 2D NMR (HMQC, HMBC, NOESY)	33
		Didemnum sp. / West Cay, Lihou Reef, off the North Queensland coast	MeOH/CH_2Cl_2	-VLC (silica gel) and RP HPLC	-HREIMS, 1D (^{1}H, ^{13}C, INEPT and NOE) and 2D NMR (XHCORRD, HMQC and COLOC)	35

**Lamellarin K triacetate (77)**	Alkaloid	*Didemnum obscurum* / Tiruchandur coast in the Gulf of Mannar, Tamilnadu, India	MeOH	-TLC, CC (silica gel), GFC-Sephadex LH-20, C18 HPLC	-UV, IR, MS, 1D (^{1}H, ^{13}C) and 2D NMR (HMQC, HMBC, NOESY)	33
**Lamellarin L triacetate (78)**	Alkaloid	*Didemnum obscurum* / Tiruchandur coast in the Gulf of Mannar, Tamilnadu, India	MeOH	-TLC, CC (silica gel), GFC-Sephadex LH-20, C18 HPLC	-UV, IR, MS, 1D (^{1}H, ^{13}C) and 2D NMR (HMQC, HMBC, NOESY)	33
**Lamellarin F (73)**	Alkaloid	*Didemnum obscurum* / Tiruchandur coast in the Gulf of Mannar, Tamilnadu, India	MeOH	-TLC, CC (silica gel), GFC-Sephadex LH-20, C18 HPLC	-UV, IR, MS, 1D (^{1}H, ^{13}C) and 2D NMR (HMQC, HMBC, NOESY)	33
**Lamellarin T diacetate (79)**	Alkaloid	*Didemnum obscurum* / Tiruchandur coast in the Gulf of Mannar, Tamilnadu, India	MeOH	-TLC, CC (silica gel, GFC-Sephadex LH-20, C18 HPLC	-UV, IR, MS, 1D (^{1}H, ^{13}C) and 2D NMR (HMQC, HMBC, NOESY)	33
**Lamellarin S (87)**	Alkaloid	*Didemnum* sp. / Wasp Island, New South Wales	Aqueous EtOH	-LLPC, and HPLC-UV-DAD	-UV, HRMS, 1D (^{1}H, ^{13}C) and 2D NMR (HMQC, HMBC, NOESY)	34
		Didemnum sp. / Australia	EtOH	LLPC, GPC-Sephadex LH-20, C18-Sep Pak, RP HPLC	-UV, HRMS, 1D (^{1}H, ^{13}C) and 2NMR (HETCOR, COLOC, COSY)	36
**Lamellarins A1-A3 (80-82)**	Alkaloid	*Didemnum* sp. / Wasp Island, New South Wales	Aqueous EtOH	-LLPC, and HPLC-UV-DAD	-UV, HRMS, 1D (^{1}H, ^{13}C) and 2D NMR (HMQC, HMBC and NOESY)	34
**Lamellarin E (59)**	Alkaloid	*Didemnum* sp. / Wasp Island, New South Wales	Aqueous EtOH	-LLPC, and HPLC-UV-DAD	-UV, HRMS, 1D (^{1}H, ^{13}C) and 2D NMR (HMQC, HMBC, NOESY)	34
		Didemnum chartaceum / Australia	MeOH	-LLPC, Sephadex LH-20, NP HPLC, RP HPLC, C18 SPE cartridge	-UV, IR, HRESMS, 1D (^{1}H, ^{13}C) and 2D NMR (COSY, HSQC, HMBC, ROESY)	32
**Lamellarin M (86)**	Alkaloid	*Didemnum* sp. / Wasp Island, New South Wales	Aqueous EtOH	-LLPC, and HPLC-UV-DAD	-UV, HRMS, 1D (^{1}H, ^{13}C) and 2D NMR (HMQC, HMBC, NOESY)	34
		Dark purple colonies of *Didemnum* sp. / South West Cay, Lihou Reef, off the North Queensland coast	$MeOH/CH_2Cl_2$	-VLC (silica gel) and RP HPLC	HREIMS, 1D (^{1}H, ^{13}C, INEPT, NOE) and 2D NMR (XHCORRD, HMQC, COLOC)	35
**Lamellarin A6 (85)**	Alkaloid	*Didemnum* sp. / Northern Rottnest Shelf, Western Australia	Aqueous EtOH	-LLPC, HPLC-UV-DAD	-UV, HRMS, 1D (^{1}H, ^{13}C) and 2D NMR (HMQC, HMBC, NOESY)	34
		Didemnum sp. / Northern Rottnest Shelf, Western Australia	EtOH	-LLP, RP HPLC	-UV, HRMS, 1D (^{1}H, ^{13}C) and 2D NMR (COSY, ROESY, HMBC).	82
**Lamellarin L (61)**	Alkaloid	Dark purple colonies of *Didemnum* sp. / South West Cay, Lihou Reef, off the North Queensland coast	$MeOH/CH_2Cl_2$	-VLC (silica gel), RP HPLC	-HREIMS, 1D (^{1}H, ^{13}C, INEPT and NOE) and 2D NMR (XHCORRD, HMQC, COLOC)	35
		Didemnum chartaceum / Australia	MeOH	-LLPC, Sephadex LH20, RP HPLC, NP HPLC, C18 SPE cartridge	-UV, IR, HRESMS,1D (^{1}H, ^{13}C) and 2D NMR, (COSY, HSQC, HMBC, ROESY)	32
		Purple encrusting *Didemnum* sp. / Indian Ocean	70% $MeOH/CHCl_3$	-LLPC, GFC (Sephadex LH-20, Spectra Gel HW-40), RP HPLC	-IR, UV, LRMS and HRMS of DEI and FAB 1D (^{1}H, ^{13}C) and 2D NMR (DQFCOSY, HMQC, HMBC, NOESY)	37

**Lamellarin β (90)**	Alkaloid	Purple encrusting *Didemnum* sp. / Indian Ocean	70% MeOH/$CHCl_3$	-LLPC, GFC (Sephadex LH20, Spectra Gel HW-40), RP HPLC	-IR, UV, LRMS, HRMS of DEI, FABMS, 1D (^{1}H, ^{13}C) and 2D NMR (DQFCOSY, HMQC, HMBC, NOESY)	37
**Lamellarin K-20-sulfate (91)**	Alkaloid	*Didemnum ternerratum* / Kingdom of Tonga	MeOH	-CC (RP, HP20 and HP20ss) and C18 HPLC	- UV, HRESIMS, 1D (1H and 13C) and 2D NMR (COSY, HMBC and ROESY).	38
**Lamellarin E-20-sulfate (92)**	Alkaloid	*Didemnum ternerratum* / Kingdom of Tonga	MeOH	-CC (RP, HP20 and HP20ss) and C18 HPLC	- UV, HRESIMS, 1D (1H and 13C) and 2D NMR (COSY, HMBC and ROESY).	38
**Lamellarin A3-20-sulfate (93)**	Alkaloid	*Didemnum ternerratum* / Kingdom of Tonga	MeOH	-CC (RP, HP20 and HP20ss) and C18 HPLC	- UV, HRESIMS, 1D (1H and 13C) and 2D NMR (COSY, HMBC and ROESY).	38
**Lamellarin B1-20-sulfate (94)**	Alkaloid	*Didemnum ternerratum* / Kingdom of Tonga	MeOH	-CC (RP, HP20 and HP20ss) and C18 HPLC	- UV, HRESIMS, 1D (1H and 13C) and 2D NMR (COSY, HMBC and ROESY).	38
**Lamellarin D-8-sulfate (95)**	Alkaloid	*Didemnum ternerratum* / Kingdom of Tonga	MeOH	-CC (RP, HP20 and HP20ss) and C18 HPLC	- UV, HRESIMS, 1D (1H and 13C) and 2D NMR (COSY, HMBC and ROESY).	38
**Lamellarin B2-20-sulfate (96)**	Alkaloid	*Didemnum ternerratum* / Kingdom of Tonga	MeOH	-CC (RP, HP20 and HP20ss) and C18 HPLC	- UV, HRESIMS, 1D (1H and 13C) and 2D NMR (COSY, HMBC and ROESY).	38
**Granulatimide (101)**	Alkaloid	*Didemnum granulatum*/ Brazil	MeOH	-CC Sephadex LH20, RP HPLC	- UV, IR, HRFABMS, 1D (^{1}H, ^{13}C, NOE) and 2D NMR (COSY, HMQC, HMBC)	40, 41
**Isogranulatimide (102)**	Alkaloid	*Didemnum granulatum* / Brazil	MeOH	-CC Sephadex LH20, RP HPLC	-UV, IR, HRFABMS, 1D (^{1}H, ^{13}C, NOE) and 2D NMR (COSY, HMQC, HMBC)	40
**6-Bromogranulatimide (104)**	Alkaloid	*Didemnum granulatum* / Brazil	MeOH followed by DMF	-FLC (Silica gel), NP HPLC	-HRFABMS, UV, IR, 1D (^{1}H, ^{13}C) and 2D NMR (HMQC, HMBC)	41
**Didemnolines A-C (105-107)**	Alkaloid	*Didemnum* sp. / Island of Rota, Northern Mariana Islands	MeOH	-FLC (Silica gel), NP HPLC	-IR, UV, HREIMS, 1D (^{1}H ,^{13}C, NOE) and 2D NMR (HMBC)	42
**Bengacarboline (113)**	Alkaloid	*Didemnum* sp. / Fiji	MeOH	-LLP, CC (Sephadex LH-20, C18 RP)	-IR, UV, FABHRMS, 1D (^{1}H, ^{13}C) and 2D NMR (COSY, TOCSY, HMQC, HMBC)	44
**Fascaplysin (114)**	Alkaloid	*Didemnum* sp. / Micronesia	MeOH/CH_2Cl_2 (1:1)	-LLP, CC (Silica gel), RP HPLC	-FABHRMS, 1D (^{1}H and ^{13}C), 2D NMR (COSY, HMQC, HMBC, NOESY)	45
		Didemnum sp. / Fiji	MeOH	-LLP, CC (Sephadex LH-20, C-18)	-IR, UV, FABHRMS, 1D (^{1}H and ^{13}C) and 2D NMR (COSY, TOCSY, HMQC HMBC)	44, 78
**3-Bromofascaplysin (115)**	Alkaloid	*Didemnum* sp. / Micronesia	MeOH/CH_2Cl_2 (1:1)	-LLP, CC (Silica gel), RP HPLC	-FABHRMS, 1D (^{1}H and ^{13}C), 2D NMR (COSY, HMQC, HMBC, NOESY)	45
**Mollamide (122)**	Cyclic peptide / cyanobactin	*Didemnum molle* / Net Reef, in the central section of the Great Barrier Reef, Australia	MeOH/CH_2Cl_2	-CC (silica gel), RP HPLC	-IR, HRESIMS, 1D (^{1}H, ^{13}C, and DEPT) and 2D NMR (COSY, HMBC NOESY), X-ray crystallographic study	46
**Mollamide C (124)**	Cyclic peptide / cyanobactin	*Didemnum molle /* Indonesia	H_2O	-VLC (Silica gel), NP and RP HPLC	-UV, IR, HRESIMS, 1D (^{1}H and ^{13}C) 2 D NMR (COSY, HMQC, ROESY, HMBC)	47

**Keenamide A (125)**	Cyclic peptide / cyanobactin	*Didemnum molle* / Indonesia	H_2O	-VLC (Silica gel), NP and RP HPLC	-UV, IR, HRESIMS, 1D (^{1}H and ^{13}C) and 2 D NMR (COSY, HMQC, ROESY, HMBC)	47
**Shishijimicins A-C (126-128)**	Polyketides / enediynes	*Didemnum proliferum* / Southern Japan	MeOH and EtOH	-LLPC, and RP HPLC	-HRFABMS, 1D (^{1}H and ^{13}C) and 2D NMR (COSY, HOHAHA, HMBC, HMQC and NOESY)	48
**Namenamicin (129)**	Polyketide / enediynes	*Didemnum proliferum* / Southern Japan	MeOH and EtOH	-LLPC, RP HPLC	-HRFABMS, 1D (^{1}H and ^{13}C) and 2D NMR (COSY, HOHAHA, HMBC, HMQC, NOESY)	48
**Plakinidine D (130)**	Alkaloid / pyrroloacridine	*Didemnum rubeum* / Island of Rota, Northern Mariana Islands	EtOH MeOH	-TLC, LLP, FLC (Silica gel)	-UV, IR, HREIMS, 1D (^{1}H, ^{13}C) and 2D NMR (COSY, INADEQUATE, HMBC)	49, 50
**Ascididemin (131)**	Alkaloid / Pyridoacridine	*Didemnum* sp. / Okinawa, Japan	MeOH	-LLP, CC (silica gel)	-UV, IR, HRFABM, EIMS,1D (^{1}H, ^{13}C, NOE) and 2D NMR (COLOC)	51
		Didemnum rubeum / The Republic of Palau	MeOH	-CC (silica gel, Sephadex LH-20)	-UV, IR, HREIMS, 1D (^{1}H, ^{13}C) and 2D NMR (COSY, HMBC)	50
		Red *Didemnum* sp. / Indonesia	MeOH	-CC (silica gel and LH-20)	-UV, IR, HREIMS, 1D (^{1}H, ^{13}C) and 2D NMR (COSY, HMBC)	50
**Methyl myristate (139)**	Fatty acid methyl ester	*Didemnum psammatodes* / Intertidal zone of the beach rocks at Flexeiras Beach, Brazil	MeOH	-CC (silica gel and LH-20), RP HPLC (C18) and GC	-IR-FT, 1D (^{1}H, ^{13}C) and 2D NMR	53
**Methyl palmitate (140)**	Fatty acid methyl ester	*Didemnum psammatodes* / Intertidal zone of the beach rocks at Flexeiras Beach, Brazil	MeOH	-CC (silica gel, LH-20), C18 HPLC, GC	-IR-FT, 1D NMR (^{1}H, ^{13}C) and 2D NMR	53
**Methyl stearate (141)**	Fatty acid methyl ester	*Didemnum psammatodes* / Intertidal zone of the beach rocks at Flexeiras Beach, Brazil	MeOH	-CC (silica gel, LH-20), RP HPLC (C18) and GC	-IR-FT, 1D NMR (^{1}H, ^{13}C) and 2D NMR	53
**Eusynstyelamide B (142)**	Bis-indole alkaloid	*Didemnum candidum* / Great Barrier Reef, Australia	CH_2Cl_2/MeOH	-Semi-preparative C18 RP HPLC	-CD, MS, 1D (^{1}H, ^{13}C) and 2D NMR (COSY, HSQC, HMBC)	54
**Vitilevuamide (143)**	Bicyclic depsipeptide	*Didemnum cuculliferum* / Namenalala Island, Fiji	MeOH	-FLC (silica gel), RP FLC, and RP HPLC	-UV, IR, HRMS, 1D (^{1}H ,^{13} C, DEPT) and 2D NMR (DQF COSY, COSY, TOCSY, HMQC, HMBC)	55
**Didemnenones C and D (144 and 145)**	Fatty acid or polyketide / yclopentenones	*Didemnum voeltzkowi* / Fringing reef, Suva Harbor, Fiji	MeOH	-LLP and CC (Sephadex LH-20)	-UV, IR, HRMS, 1D NMR (^{1}H, ^{13}C)	56
**Didemnilactones A and B (146 and 147)**	Fatty acid / 10-membered ring lactone	*Didemnum moseleyi* / Ago Bay, Mie Prefecture, Japan	MeOH	-CC (silica gel and alumina), RP HPLC	-UV, IR, HRESIMS, 1D (^{1}H, ^{13}C) and 2D NMR (COSY, HMQC)	57
**Neodidemnilactone (148)**	Fatty acid	*Didemnum moseleyi* / Ago Bay, Mie Prefecture, Japan	MeOH	-CC (silica gel and alumina), RP HPLC	-UV, IR, HRESIMS, 1D (^{1}H, ^{13}C) and 2D NMR (COSY, HMQC)	57
**Cyclodidemnamide (149)**	Cyclic peptide / cyanobactin	*Didemnum molle* / Philippines	30% CH_2Cl_2 in MeOH	-LLP, CC (silica gel, LH-20), RP HPLC	-UV, IR, GC-MS, 1D (^{1}H, ^{13}C) and 2D NMR (COSY, HMBC, HMQC, ROSY)	58

**Comoramides A and B (152 and 153)**	Cyclic peptide / cyanobactin	*Didemnum molle* / Mayotte	CHC1$_3$/MeOH (1:2)	-VLC (silica gel)	-FT-IR, HRMS, 1D (^{1}H, ^{13}C) and 2D NMR (COSY, TCOSY, HMBC, NOESY)	59
**Mayotamides A and B (150 and 151)**	Cyclic peptide / cyanobactin	*Didemnum molle* / Mayotte	CHC1$_3$/MeOH (1:2)	-VLC (silica gel)	-FT-IR, HRMS, 1D (^{1}H, ^{13}C) and 2D NMR (COSY, TCOSY, HMBC, NOESY)	59
**Caledonin (154)**	Peptide	*Didemnum rodriguesi* / Baie des Citrons, New Caledonia	MeOH	-LLP, CC (Silica gel, Sephadex LH-20), RP HPLC	-UV, IR, HRMS, FABMS, 1D (^{1} H, ^{13}C, DEPT) and 2D NMR (DQF-COSY, TOCSY, HMQC, ROSY	60
**Hexamollamide (161)**	Cyclic peptide / cyanobactin	*Didemnum molle* / Okinawa, Japan	MeOH	-LLP, RP CC (ODS), RF HPLC and Chiral HPLC	-ESIMS, 1D (^{1}H, ^{13}C) and 2D NMR (COSY, HSQC, HMBC)	62

Table S2. Compounds with antimicrobial activities.

Compound Name	Chemical Class	Source organism / Location	Solvent of Extraction	Purification of the Compounds	Structural Determination	Ref.
**Rodriguesines A and B (162 and 163)**	Peptide / diketopiperazine	*Didemnum* sp. / Salvador, Brazil	MeOH	-CC (C18, Sephadex LH-20), RP HPLC	-UV, IR, HRESIMS, 1D NMR (^{1}H, ^{13}C) and 2D NMR (COSY, HMQC, HMBC)	63
**Didemnaketals A and B (179 and 180)**	Polyketide / spiroketal	*Didemnum* sp. / Auluptagel Island, Palau	CH_2Cl_2/MeOH (1:1)	-Sephadex LH-20, RP HPLC	-IR, HRFABMS, 1D (^{1}H, ^{13}C) and 2D NMR (COSY, HMQC, HMBC)	69
**Didemnaketals D and E (166 and 167)**	Polyketide / spiroketal	*Didemnum* sp. / Sharm El-Sheikh, Red Sea, Egypt	CH_2Cl_2/MeOH (1:1)	-LLP, VLC (Silica gel), Sephadex LH20, RP HPLC	-UV, IR, HRESIMS, 1D (^{1}H, ^{13}C, DEPT) and 2D NMR (COSY, ROESY, NOESY, TOCSY, HSQC, HMBC)	64
**Didemnaketals F and G (173 and 174)**	Polyketide / spiroketal	*Didemnum* sp. / Red Sea, Egypt	CH_2Cl_2/MeOH (1:1)	-VLC, Sephadex LH-20, C18 HPLC	-IR, UV, HRESIMS, 1D (^{1}H, ^{13}C, DEPT) and 2D (COSY, ROESY, NOESY, TOCSY, HSQC, HMBC)	66
**Mollamides E and F (186-187)**	Cyclic peptide / cyanobactin	*Didemnum molle* / New Britain, Papua New Guinea	MeOH	-CC (HP20SS resin), FLC (C18), RP HPLC	-UV, IR, HRESIMS, 1D (^{1}H, ^{13}C) and 2D NMR (gCOSY, HMBC, ROESY)	72
**Molleurea A (5)**	Alkaloid / phenylethylamine	*Didemnum molle* / New Britain, Papua New Guinea	MeOH	-CC (HP20SS resin), FLC (C18) and RP HPLC	-IR, UV, HRESIMS, 1D NMR (^{1}H, ^{13}C) 2DNMR (gCOSY, HMBC, ROESY)	72
		Didemnum molle / Mayotte	MeOH/CH_2Cl_2 (1:1)	-Kupchan solvent partition procedure, CC (Sephadex LH-20), RP HPLC	-UV, IR HRMS, LCMS, 1D (^{1}H, ^{13}C, DEPT) and 2D (COSY, TOCSY, HSQC, HMQC, HMBC, ROESY)	17
**3,5-Diiodo-4-methoxyphenethylamine (27)**	Alkaloid / phenylethylamine	*Didemnum* sp. / Northwest end of Cocos Lagoon, Guam, United States	Organic solvents	-Crystallization from a $CHC1_3$ extract	-HRMS, 1D NMR (^{1}H, ^{13}C)	52
		Didemnum rubeum / Republic of Palau	EtOH MeOH	-LLP, and FLC (Silica gel)	-UV, IR, HREIMS, 1D (^{1}H, ^{13}C) and 2D NMR (COSY, INADEQUATE, HMBC)	49, 50
**Kakelokelose homopolysaccharide (188)**	Carboyhydrate / sulfated mannose	*Didemnum molle* / Pohnpei, Micronesia	95% EtOH	-Ultrafiltration membranes are expressed as Molecular Weight Cutoff (MWCO)	-FT-IR, 1D (^{1}H, ^{13}C, DEPT) and 2D NMR (COSY, HMQC)	73
**Cyclodidemniserinol trisulfate (182)**	Fatty acid / serinolipid	*Didemnum guttatum* / Ngerchaol Island, Palau	MeOH	-Purified by repeated reversed phase chromatography	-UV, IR, HRMS, 1D (^{1}H, ^{13}C) and 2D NMR (COSY, HSQC-TOCSY, HMBC)	71
**(*R*)-(*E*)-1-Aminotridec-5-en-2-ol (191)**	Alkaloid	*Didemnum* sp. / Great Barrier Reef, Australia	MeOH	-Kupchan solvent partition procedure, CC (Sephadex LH-20), FLC (C18) RP HPLC	-UV, IR, MS, and 1D NMR (^{1}H, ^{13}C, DEPT) 2D NMR (COSY,HMQC)	75
**Divamides A and B (189 and 190)**	Cyclic peptide / lanthipeptide	*Didemnum molle* / Eastern Fields, Papua New Guinea	MeOH / metagenomics method	-HP20, HPLC (C18)	-UV, MS, 1D NMR (1H, 13C) 2D NMR (COSY, TOCSY, HMQC, HMBC, ROESY, NOESY), metagenome sequencing	74

Table S3. Compounds with antiprotozoal activities.

Compound Name	Chemical Class	Source organism / Location	Solvent of Extraction	Purification of the compounds	Structural Determination	Ref.
**Lepadins D-F (194-196)**	Alkaloid / decahydroquinoline	*Didemnum* sp. / Stanley Reef, the Great Barrier Reef, Australia	Acetone	-TLC, NP VLC, NP HPLC	-HREIMS, 1D (^{1}H, ^{13}C) and 2D NMR (COSY, HMQC, HMBC, ROESY, NOE)	76
**Albopunctatone (197)**	Polyketide / anthrone-anthraquinone	*Didemnum albopunctatum* / Swain Reefs in the Southern Great Barrier Reef, Australia	MeOH	-NP HPLC, RP HPLC	-UV, IR, MS, 1D (^{1}H, ^{13}C) and 2D NMR (COSY, HSQC, HMBC)	77
**Homofascaplysin A (199)**	Alkaloid	*Didemnum* sp. / Fiji	MeOH	-LLP, CC (Sephadex LH-20, C-18 RP)	-UV, IR, FABHRMS, ECD, 1D (^{1}H and ^{13}C) and 2D NMR (COSY, TOCSY, HMQC, HMBC)	78
**Mollamide B (123)**	Cyclic peptide / cyanobactin	*Didemnum molle* / Indonesia	H_2O	-VLC (Silica gel), NP and RP HPLC	-UV, IR, HRESIMS, 1D (^{1}H and ^{13}C) 2 D NMR (COSY, HMQC, HMBC, ROESY)	47
**Didemnidine B (202)**	Alkaloid / indole spermidine	*Didemnum* sp. / New Zealand	MeOH	-FLC (C8 and C18 RP), Sephadex LH-20, RP HPLC	-UV, IR, HRMS, 1D (^{1}H, ^{13}C) and 2D NMR (COSY, HSQC, HMBC)	79

Table S4. Compounds with antidiabetic activities.

Compound Name	Chemical Class	Source organism / Location	Solvent of Extraction	Purification of the compounds	Structural Determination	Ref.
***N,N'*-Diphenethyl-urea (11)**	Alkaloid / phenylethlyamine	*Didemnum mole /* Okinawa, Japan	MeOH	-LLP, fractionation using ODS RP	-UV, IR, MS, 1D (^{1}H, ^{13}C)	80
		Didemnum ternatanum / Urukthapal, Palau	MeOH	-CC (Silica gel, Sephadex LH-20)	-IR, HRMS, EIMS, 1D NMR (^{1}H, ^{13}C)	16
		Didemnum molle / Mayotte	MeOH/CH_2Cl_2 (1:1)	-Kupchan solvent partition procedure, CC (Sephadex LH-20), RP HPLC	-UV, IR, HRMS, LCMS, 1D (^{1}H, ^{13}C, DEPT) and 2D NMR (COSY, TOCSY, HSQC, HMQC, HMBC, ROESY)	17

Table S5. Compounds that affect the central nervous system.

Compound Name	Chemical Class	Source organism / Location	Solvent of Extraction	Purification of the compounds	Structural Determination	Ref.
**Ningalin B-D (204-2067)**	Alkaloid	*Didemnum sp.* / Northern Rottnest Shelf, Western Australia	EtOH	-LLP, RP HPLC	-UV, HRMS, 1D (^{1}H, ^{13}C) and 2D NMR (COSY, ROESY, HMBC)	82
		Didemnum sp. / near Ningaloo Reef, Western Australia	70% MeOH in CH_2Cl_2	-LLP, GFC (Sephadex LH-20), C18 HPLC, GPC (Spectra Gel HW-40)	-UV, IR, HRFABMS, 1D (^{1}H, ^{13}C) and 2D NMR (COSY, HMQC, HMBC, NOESY)	81
**Ningalin E-G (207-209)**	Alkaloid	*Didemnum sp.* / Northern Rottnest Shelf, Western Australia	EtOH	-LLP, RP HPLC	-UV, HRMS, 1D (^{1}H, ^{13}C) and 2D NMR (COSY, ROESY, HMBC)	82
**Lamellarin G (60)**	Alkaloid	*Didemnum* sp. / Northern Rottnest Shelf, Western Australia	EtOH	-LLP, RP HPLC	-UV, HRMS, 1D (^{1}H, ^{13}C) and 2D NMR (COSY, ROESY, HMBC)	82
		Didemnum sp. / Northern Rottnest Shelf, Western Australia	Aqueous EtOH	-LLPC, HPLC-UV-DAD	-HRMS, UV, 1D (^{1}H, ^{13}C) and 2D NMR (COSY, HMBC, ROESY).	34
		Didemnum chartaceum / Australia	MeOH	-LLPC, Sephadex LH-20, NP HPLC, RP HPLC, C18 SPE cartridge	-HRESMS, UV, IR, 1D (^{1}H, ^{13}C) and 2D NMR (COSY, HSQC, HMBC, ROESY)	32
		Purple encrusting *Didemnum* sp. / the Indian Ocean	70% MeOH/$CHCl_3$	-LLPC, GFC (Sephadex LH-20 and Spectra Gel HW-40), RP HPLC	-IR, UV, LRMS, HRMS of DEI, FABMS, 1D (^{1}H, ^{13}C) and 2D NMR. (DQFCOSY, HMQC HMBC, NOESY)	37
**Lamellarin Z (62)**	Alkaloid	*Didemnum sp.* / Northern Rottnest Shelf, Western Australia	EtOH	-LLP, RP HPLC	-UV, HRMS, 1D (^{1}H, ^{13}C) and 2D NMR (COSY, ROESY, HMBC)	82
		Didemnum sp. / Northern Rottnest Shelf, Western Australia	Aqueous EtOH	-LLPC, and HPLC-UV-DAD	-HRMS, UV, 1D (^{1}H, ^{13}C) and 2D NMR (COSY, HMBC, ROESY)	34
		Didemnum chartaceum / Australia	MeOH	-LLPC, Sephadex LH-20, NP HPLC, RP HPLC, C-18 SPE cartridge	-HRESMS, UV, IR, 1D (^{1}H, ^{13}C) and 2D NMR (COSY, HSQC, HMBC, ROESY)	32
**Lepadin I (210)**	Alkaloid	*Didemnum* sp. / Bahamas	70% MeOH in CH_2Cl_2	-LLP, CC (Silica), RP HPLC, C-18 SPE cartridge	-HRMS, ESIMS IR, UV, ECCD, 1D (^{1}H, ^{13}C) and 2D NMR (COSY, HMBC, HSQC, NOESY)	83

Table S6. Compounds without any reported biological activities.

Compound Name	Chemical Class	Source organism / Location	Solvent of Extraction	Purification of the compounds	Structural Determination	Ref.
**Lepadin J and K (211 and 212)**	Alkaloid	*Didemnum* sp. / Bahamas	MeOH/CH_2Cl_2 (1:1)	-LLP, CC (Silica), RP HPLC, C18 SPE cartridges	-UV, ESIMS IR, HRMS, ECCD, 1D (^{1}H, ^{13}C) and 2D NMR (COSY, HSQC, HMBC)	[83]
**1,8-Dihydroxy-9,10 anthraquinone (199)**	Polyketide / anthraquinone	*Didemnum albopunctatum /* Australia	MeOH	-NP HPLC, RP HPLC	-UV, IR, MS, 1D (^{1}H, ^{13}C) and 2D NMR (COSY, HSQC, HMBC)	[77]
**Salvadenosine (43)**	Nucleoside	*Didemnum* sp. / Bahamas	MeOH	-LLP, RP HPLC	-HRMS and 1D (^{1}H, ^{13}C), 2D NMR (COSY, HSQC, HMBC)	[26]
**Lamellarin A4 and A5 (83 and 84)**	Alkaloid	*Didemnum* sp. / Wasp Island, New South Wales, Australia	MeOH	-LLPC, HPLC-UV-DAD	-UV, HRMS, 1D (^{1}H, ^{13}C) and 2D NMR (HMQC, HMBC, NOESY)	[34]
**Lamellarin C (58)**	Alkaloid	*Didemnum* sp. / Wasp Island, New South Wales, Australia	Aqueous EtOH	-LLPC, HPLC-UV DAD	-UV, HRMS, 1D (^{1}H, ^{13}C) and 2D NMR (COSY, HMBC, ROESY)	[34]
		Didemnum chartaceum / Australia	MeOH	-LLPC, Sephadex LH-20, NP and RP HPLC, C18-SPE cartridge	-HRESMS, UV, IR, 1D (^{1}H, ^{13}C) and 2D NMR (COSY, HSQC, HMBC, ROESY)	[32]
		Didemnum sp. / South West Cay, Lihou Reef, off the North Queensland coast, Australia	MeOH/CH_2Cl_2	-VLC (silica gel), RP HPLC	-HREIMS, 1D (^{1}H, ^{13}C, INEPT and NOE) and 2D NMR (XHCORRD, HMQC, COLOC)	[35]
**Lamellarin T (88)**	Alkaloid	*Didemnum* sp. / Wasp Island, New South Wales, Australia	Aqueous EtOH	-LLPC, HPLC-UV DAD	-UV, HRMS, 1D NMR (^{1}H, ^{13}C) and 2D NMR, (COSY, HMBC, ROESY)	[34]
**Lamellarin X (89)**	Alkaloid	*Didemnum* sp. / Wasp Island, New South Wales, Australia	Aqueous EtOH	-LLPC, HPLC-UV-DAD	-HRMS, UV, 1D (^{1}H, ^{13}C) and 2D NMR (COSY, HMBC, ROESY)	[34]
**Lamellarin B 20-sulfate (63)**	Alkaloid	*Didemnum chartaceum /* Australia	MeOH	-LLPC, Sephadex LH-20, NP HPLC, RP HPLC, C18 SPE cartridge	-UV, IR, HRESMS, 1D NMR (^{1}H, ^{13}C) and 2D NMR (COSY, HSQC, HMBC, ROESY)	[32]
**Lamellarin C 20-sulfate (64)**	Alkaloid	*Didemnum chartaceum /* Australia	MeOH	-LLPC, Sephadex LH-20, NP HPLC, RP HPLC, C18 SPE cartridge	-UV, IR, HRESMS, 1D (^{1}H, ^{13}C) and 2D NMR (COSY, HSQC, HMBC, ROESY)	[32]
**Lamellarin L 20-sulfate (65)**	Alkaloid	*Didemnum chartaceum /* Australia	MeOH	-LLPC, Sephadex LH-20, NP HPLC, RP HPLC, C18 SPE cartridge	-UV, IR, HRESMS, 1D (^{1}H, ^{13}C) and 2D NMR (COSY, HSQC, HMBC, ROESY)	[32]

**Lamellarin A (56)**	Alkaloid	*Didemnum chartaceum* / Australia	MeOH	-LLPC, Sephadex LH-20, NP HPLC, RP HPLC, C18 SPE cartridge	-UV, IR, HRESMS, 1D (^{1}H, ^{13}C) and 2D NMR (COSY, HSQC, HMBC, ROESY)	32
		Didemnum sp. / South West Cay, Lihou Reef, off the North Queensland coast	MeOH/CH_2Cl_2	-VLC (silica gel), RP HPLC	-HREIMS, 1D (^{1}H, ^{13}C, INEPT, NOE) and 2D NMR (XHCORRD, HMQC, COLOC)	35
**Lamellarin B (57)**	Alkaloid	*Didemnum chartaceum* / Australia	MeOH	-LLPC, Sephadex LH-20, NP and RP HPLC, C-18 SPE cartridge	-UV, IR, HRESMS, 1D (^{1}H, ^{13}C) and 2D NMR (COSY, HSQC, HMBC, ROESY)	32
		Dark purple encrusting colonies of *Didemnum* sp. / South West Cay, Lihou Reef, off the North Queensland coast	MeOH/CH_2Cl_2	-VLC (silica gel), RP HPLC	-HREIMS, 1D (^{1}H, ^{13}C, INEPT, NOE) and 2D NMR (XHCORRD, HMQC, COLOC)	35
**Lamellarin D triacetate (67)**	Alkaloid	*Didemnum chartaceum* / Australia	MeOH	-LLPC, Sephadex LH-20, NP HPLC, RP HPLC, C18 SPE cartridge	-UV, IR, HRESMS, 1D (^{1}H, ^{13}C) and 2D NMR (COSY, HSQC, HMBC, ROESY).	32
		Dark purple encrusting colonies of *Didemnum* sp. / South West Cay, Lihou Reef, off the North Queensland coast	MeOH/CH_2Cl_2	-VLC (silica gel), RP HPLC	-HREIMS, 1D (^{1}H ,^{13}C, INEPT, NOE) and 2D NMR (XHCORRD, HMQC, COLOC)	35
**Lamellarin N triacetate (68)**	Alkaloid	*Didemnum chartaceum* / Australia	MeOH	-LLPC, Sephadex LH-20, NP HPLC, RP HPLC and SPE cartridge packed with C18	-UV, IR, HRESMS, 1D (^{1}H, ^{13}C) and 2D NMR (COSY, HSQC, HMBC, ROESY)	32
**Ningalin A (203)**	Alkaloid	*Didemnum* sp. / near Ningaloo Reef, Western Australia	70% MeOH in CH_2Cl_2	-LLP, GFC (Sephadex LH-20), C18 HPLC, GPC (Spectra Gel HW-40)	-UV, IR, HRFABMS 1D (^{1}H, ^{13}C) and 2D NMR (COSY, HMQC, HMBC, NOESY)	81
**Didemnimide A (97)**	Alkaloid	*Didemnum conchyliatum* / Sweetings Cay, Bahamas	MeOH/CH_2Cl_2 (1:1)	-VLC, NP CC, C18 RP CC, RP HPLC	-UV, IR, HREIMS, 1D (^{1}H, ^{13}C) and 2D NMR (HMQC, HMBC), X-ray analysis	39
		Didemnum granulatum / Brazil	MeOH	-CC, Sephadex LH20, RP HPLC	-UV, IR, HRFABMS, 1D (^{1}H ,^{13}C, NOE) and 2D NMR (COSY, HMQC, HMBC)	40
**Didemnimide B (98)**	Alkaloid	*Didemnum conchyliatum* / Sweetings Cay, Bahamas	MeOH/CH_2Cl_2 (1:1)	-VLC, NP CC, C18 RP, CC, RP HPLC	-HREIMS, UV, IR, 1D (^{1}H, ^{13}C) and 2D NMR (HMQC, HMBC), X-ray analysis	39
**Didemnimide C (99)**	Alkaloid	*Didemnum conchyliatum* / Sweetings Cay, Bahamas	MeOH/CH_2Cl_2 (1:1)	-VLC, NP CC, C18 RP, RP HPLC	-HREIMS, UV, IR, 1D (^{1}H, ^{13}C) and 2D NMR (HMQC, HMBC), X-ray analysis	39
**Didemnimide D (100)**	Alkaloid	*Didemnum conchyliatum* / Sweetings Cay, Bahamas	MeOH/CH_2Cl_2 (1:1)	-VLC, NP CC, C18 RP, RP HPLC	-UV, IR, HREIMS, 1D (^{1}H, ^{13}C) and 2D NMR (HMQC, HMBC), X-ray analysis	39

		Didemnum conchyliatum / Sweetings Cay, Bahamas	MeOH	-CC Sephadex LH20, RP HPLC	-UV, IR, HRFABMS, 1D (^{1}H, ^{13}C, NOE) and 2D NMR (COSY, HMQC, HMBC)	40
**Didemnimide E (103)**	Alkaloid	*Didemnum granulatum* / Brazil	MeOH	-CC Sephadex LH20, RP HPLC	-UV, IR, HRFABMS, 1D (^{1}H,^{13}C NOE) and 2D NMR (COSY, HMQC, HMBC)	40
***N*-Acetyl-rodriguesines A and B (164 and 165)**	Peptide / diketopiperazine	*Didemnum* sp. / Salvador, Brazil	MeOH	-LLP, CC (Sephadex LH-20, C18), RP HPLC	-IR, UV, HRESIMS, 1D (^{1}H, ^{13}C) and 2D NMR (COSY, HMQC, HMBC)	63
**Didemniserinolipids A-C (183 and 185)**	Fatty acid / serinolipid	*Didemnum* sp. / Sulawesi Island, Indonesia	MeOH	-LLPC, VLC (silica gel), RP HPLC	-UV, IR, HRESIMS, 1D (^{1}H, ^{13}C and DEPT) and 2D NMR (1H-1H COSY, TOCSY, HMQC and ROESY)	70
**Didemnoline D (108)**	Alkaloid	*Didemnum* sp. / Near Rota, Northern Mariana Islands	MeOH	-FLC (Silica gel), NP HPLC	-UV, IR, HREIMS, 1D (^{1}H ,^{13}C and NOE) and 2D NMR (HMBC)	42
**Eudistomin O (109)**	Alkaloid	*Didemnum* sp. / Near Rota, Northern Mariana Islands	MeOH	-FLC (Silica gel), NP HPLC	-UV, IR, HREIMS, 1D (^{1}H ,^{13}C, NOE) and 2D NMR (HMBC)	42
**β-Carboline (110)**	Alkaloid	*Didemnum* sp. / Near Rota, Northern Mariana Islands	MeOH	-FLC (Silica gel), NP HPLC	-UV, IR, HREIMS, 1D (^{1}H ,^{13}C, NOE) and 2D NMR (HMBC)	42
**2-(2′,4′-Dibromophenoxy)-3,5-dibromophenol (111)**	Phenolic ether	*Didemnum* sp. / Near Rota, Northern Mariana Islands	MeOH	-FLC (Silica gel), NP HPLC	-UV, IR, HREIMS, 1D (^{1}H ,^{13}C, NOE) and 2D NMR (HMBC)	42
**β-Carboline dimers (112)**	Alkaloid	*Didemnum* sp. / Sykes Reef in the Capricorn Bunker Group of the southern Great Barrier Reef, Australia	Hexane followed by EtOAc	-CC (Silica gel)	-UV, IR, MS, 1D NMR (^{1}H, ^{13}C)	43
**3-Bromohomo-fascaplysin A (200)**	Alkaloid	*Didemnum* sp. / Fiji	MeOH	-LLP, CC (Sephadex LH-20, C18 RP)	-UV, IR, FABHRMS, ECD, 1D (^{1}H, ^{13}C) and 2D NMR (COSY, TOCSY, HMQC, HMBC)	78
**Homofascaplysin B-1 (116)**	Alkaloid	*Didemnum* sp. / Indonesia	MeOH/CH_2Cl_2 (1:1)	-LLP, FLC (Silica gel), RP HPLC	-FABHRMS, 1D (^{1}H, ^{13}C), 2D NMR (COSY, HMQC, HMBC, NOESY)	45
**3-Bromohomo-fascaplysin B (117)**	Alkaloid	*Didemnum* sp. / Indonesia	MeOH/CH_2Cl_2 (1:1)	-LLP, FLC (Silica gel), RP HPLC	-FABHRMS, 1D (^{1}H, ^{13}C) and 2D NMR (COSY, HMQC, HMBC, NOESY)	45
**Homofascaplysin C (118)**	Alkaloid	*Didemnum* sp. / Indonesia	MeOH/CH_2Cl_2 (1:1)	-LLP, FLC (Silica gel), RP HPLC	-FABHRMS, 1D (^{1}H, ^{13}C) and 2D NMR (COSY, HMQC, HMBC, NOESY)	45
**3-Bromohomo-fascaplysin B-1 (119)**	Alkaloid	*Didemnum* sp. / Indonesia	MeOH/CH_2Cl_2 (1:1)	-LLP, FLC (Silica gel), RP HPLC	-FABHRMS, 1D (^{1}H, ^{13}C) and 2D NMR (COSY, HMQC, HMBC, NOESY)	45
**3-Bromohomo-fascaplysin C (120)**	Alkaloid	*Didemnum* sp. / Indonesia	MeOH/CH_2Cl_2 (1:1)	-LLP, FLC (Silica gel), RP HPLC	-FABHRMS, 1D (^{1}H, ^{13}C) and 2D NMR (COSY, HMQC, HMBC and NOESY)	45
**Homofascaplysate A (121)**	Alkaloid	*Didemnum* sp. / Indonesia	MeOH/CH_2Cl_2 (1:1)	-LLP, FLC (Silica gel), RP HPLC	-FABHRMS, 1D (^{1}H, ^{13}C) and 2D NMR (COSY, HMQC, HMBC, NOESY)	45

**Didemnaketal C (181)**	Polyketide / spiroketals	*Didemnum* sp. / Auluptagel Island, Palau	$MeOH/CH_2Cl_2$ (1:1)	-Sephadex LH-20, RP HPLC	-UV, IR, HRFABMS, 1D NMR (^1H, ^{13}C)	68
**Mollecarbamates A-D (1−4)**	Phenylethylamine	*Didemnum molle* / Mayotte	$MeOH/CH_2Cl_2$ (1:1)	-Kupchan solvent partition procedure, CC (Sephadex LH-20), RP HPLC	-UV, IR, HRMS, LCMS, 1D (^1H, ^{13}C, DEPT) 2D (COSY, TOCSY, NOESY, HSQC, HMBC)	17
**Molleurea B-E (5−8)**	Phenylethylamine	*Didemnum molle* / Mayotte	$MeOH/CH_2Cl_2$ (1:1)	-Kupchan solvent partition procedure, CC (Sephadex LH-20), RP HPLC	-UV, IR, HRMS, LCMS, 1D (^1H, ^{13}C, DEPT) and 2D NMR (COSY, NOESY, HMBC)	17
**Molledihydroisoquinolone (10)**	Phenylethylamine	*Didemnum molle* / Baie des Assassins, Madagascar	$MeOH/CH_2Cl_2$ (1:1)	-Kupchan solvent partition procedure, CC (Sephadex LH-20), RP HPLC	-UV, IR, HRMS, LCMS, 1D (^1H, ^{13}C, DEPT) and 2D NMR (COSY, TOCSY, HSQC, HMQC, HMBC, ROESY)	17
**Molleurea E (9)**	Phenylethylamine	*Didemnum molle* / New Britain, Papua New Guinea	MeOH	-CC (HP20SS resin), C18 FLC, RP HPLC	-UV, IR, HRESIMS, 1D NMR (^1H ,^{13}C) and 2D NMR (gCOSY, HMBC and ROESY)	72
**Antatollamides A and B (14 and 15)**	Cyclic peptide / cyanobactin	*Didemnum molle* / Pohnpei	$MeOH/CH_2Cl_2$ (1:1)	-LLP, FLC (Silica gel), RP HPLC	-UV, FTIR, HRMS, 1D (^1H ,^{13}C, NOE) and 2D NMR (COSY, TOCSY, HSQC, HMBC, NOESY)	19
**Didemnidine A (201)**	Alkaloid	*Didemnum* sp. / New Zealand	MeOH	-FLC (C8, C18), Sephadex LH-20, RP HPLC	-UV, IR, HRMS, 1D (^1H, ^{13}C) and 2D NMR (COSY, HSQC, HMBC)	79
**Didemnaceride A and B (168 and 169)**	Fatty acid / glyceride	*Didemnum* sp. / Red Sea, Egypt	$CH_2Cl_2/MeOH$ (1:1)	-LLP, VLC, CC (Silica gel), CC (RP-C18)	-UV, IR, HR-ESI-MS, GC-MS, 1D (^1H, ^{13}C) and 2D NMR (COSY, HSQC, HMBC)	65
**24-Ethyl-25-hydroxycholesterol (170)**	Steroid	*Didemnum* sp. / Red Sea, Egypt	$CH_2Cl_2/MeOH$ (1:1)	-LLP, VLC, CC (Silica gel), CC (RP-C18)	-UV, IR, HR-ESI-MS, GC-MS, 1D (^1H, ^{13}C) and 2D NMR (COSY, HSQC, HMBC)	65
**Cholest-6-en-3,5,8-triol (171)**	Steroid	*Didemnum* sp. / Red Sea, Egypt	$CH_2Cl_2/MeOH$ (1:1)	-LLP, VLC, CC (Silica gel), CC (RP-C18)	-UV, IR, HR-ESI-MS, GC-MS, 1D (^1H, ^{13}C) and 2D NMR (COSY, HSQC, HMBC)	65
**Cholestane-3β,5α,6β-26-tetrol (172)**	Steroid	*Didemnum* sp. / Red Sea, Egypt	$CH_2Cl_2/MeOH$ (1:1)	-LLP, VLC, CC (Silica gel), CC (RP-C18)	-UV, IR, HR-ESI-MS, GC-MS, 1D (^1H, ^{13}C) and 2D NMR (COSY, HSQC, HMBC)	65
**Minalemines A-F (155-160)**	Peptide / guanidine	*Didemnum rodriguesi* / Baie des Citrons, Noumea, New Caledonia	MeOH	-LLP, CC (Sephadex LH-20), RP HPLC	-UV, IR, HRFABMS, 1D (^1H, ^{13}C, DEPT) and 2D NMR (COSY, TOCSY, HMQC, HMBC)	61
**Cholesterol (132)**	Steroid	*Didemnum psammatodes* / Intertidal zone of the beach rocks at Flexeiras Beach, Brazil	MeOH	-CC (silica gel, Sephadex LH-20), C18 HPLC, GC	-IR-FT, 1D (^1H and ^{13}C) and 2D NMR	53
**Campesterol (133)**	Steroid	*Didemnum psammatodes* / Intertidal zone of the beach rocks at Flexeiras Beach, Brazil	MeOH	-CC (silica gel, Sephadex LH-20), C18 HPLC, GC	-IR-FT, 1D (^1H and ^{13}C) and 2D NMR	53

**Stigmasterol (18)**	Steroid	*Didemnum psammatodes* / Intertidal zone of the beach rocks at Flexeiras Beach, Brazil	MeOH	-CC (silica gel, Sephadex LH-20), C18 HPLC, GC	-IR-FT, 1D (^{1}H and ^{13}C) and 2D NMR	[53]
**β-Sitosterol (134)**	Steroid	*Didemnum psammatodes* / Intertidal zone of the beach rocks at Flexeiras Beach, Brazil	MeOH	-CC (silica gel, Sephadex LH-20), C18 HPLC, GC	-IR-FT, 1D (^{1}H and ^{13}C) 2D NMR	[53]
**Palmitic acid (135)**	Fatty acid	*Didemnum psammatodes* / Intertidal zone of the beach rocks at Flexeiras Beach, Brazil	MeOH	-CC (silica gel, Sephadex LH-20), C18 HPLC, GC	-IR-FT, 1D (^{1}H and ^{13}C) and 2D NMR	[53]
**Stearic acid (136)**	Fatty acid	*Didemnum psammatodes* / Intertidal zone of the beach rocks at Flexeiras Beach, Brazil	MeOH	-CC (silica gel, Sephadex LH-20), C18 HPLC, GC	-IR-FT, 1D (^{1}H, ^{13}C) and 2D NMR	[53]
**1,2-Propanediol, 3-(heptadecyloxy) (137)**	Fatty acid / glyceryl ether	*Didemnum psammatodes* / Intertidal zone of the beach rocks at Flexeiras Beach, Brazil	MeOH	-CC (silica gel, Sephadex LH-20), C18 HPLC, GC	-IR-FT, 1D (^{1}H, ^{13}C) and 2D NMR	[53]
**Batyl alcohol (19)**	Fatty acid / glyceryl ether	*Didemnum psammatodes* / Intertidal zone of the beach rocks at Flexeiras Beach, Brazil	MeOH	-CC (silica gel, Sephadex LH-20), C18 HPLC, GC	-IR-FT, 1D (^{1}H, ^{13}C) and 2D NMR	[53]
**1,2-Propanediol,3[(methyloctadecyl)oxy] (138)**	Fatty acid / glyceryl ether	*Didemnum psammatodes* / Intertidal zone of the beach rocks at Flexeiras Beach, Brazil	MeOH	-CC (silica gel, Sephadex LH-20), C18 HPLC, GC	-IR-FT, 1D (^{1}H and ^{13}C) and 2D NMR	[53]
**Thymidine (21)**	Nucleoside	*Didemnum psammatodes* / Intertidal zone of the beach rocks at Flexeiras Beach, Brazil	MeOH	-CC (silica gel, Sephadex LH-20), C18 HPLC, GC	-IR-FT, 1D (^{1}H and ^{13}C) and 2D NMR	[53]
		Didemnum psammatodes / Shores of Ceará State, Brazil	MeOH	-CC (silica gel, Sephadex LH-20)	-UV, MS, GC-MS, 1D (^{1}H, ^{13}C) and 2D NMR (COSY, HSQC, HMBC)	[20]

**2′-Deoxyguanosine (23)**	Nucleoside	*Didemnum psammatodes* / Intertidal zone of the beach rocks at Flexeiras Beach, Brazil	MeOH	-CC (silica gel, Sephadex LH-20), C18 HPLC, GC	-IR-FT, 1D (^{1}H, ^{13}C) and 2D NMR	[53]
		Didemnum psammatodes / Shores of Ceará state.	MeOH	-CC (silica gel and Sephadex LH-20)	-UV, MS, GC-MS, 1D NMR (^{1}H, ^{13}C) and 2D NMR (COSY, HSQC, HMBC)	[20]
**2’-Deoxyuridine (20)**	Nucleoside	*Didemnum psammatodes* / Shores of Ceará State, Brazil	MeOH	-CC (silica gel, Sephadex LH-20)	-UV, MS, GC-MS, 1D (^{1}H, ^{13}C) and 2D NMR (COSY, HSQC, HMBC)	[20]
**2’-Deoxyinosine (22)**	Nucleoside	*Didemnum psammatodes* / Shores of Ceará State, Brazil	MeOH	-CC (silica gel and Sephadex LH-20)	-UV, MS, GC-MS, 1D (^{1}H, ^{13}C) and 2D (COSY, HSQC, HMBC)	[20]
**Cholestanol (16)**	Steroid	*Didemnum psammatodes* / Shores of Ceará State, Brazil	MeOH	-CC (silica gel, Sephadex LH-20)	-UV, MS, GC-MS, 1D (^{1}H, ^{13}C) and 2D NMR (COSY, HSQC, HMBC)	[20]
**Cholestanone (17)**	Steroid	*Didemnum psammatodes* / Shores of Ceará State, Brazil	MeOH	-CC (silica gel, Sephadex LH-20)	-UV, MS, GC-MS, 1D (^{1}H, ^{13}C) and 2D NMR (COSY, HSQC, HMBC)	[20]
**16-Epi-18-acetyl herdmanine D (24)**	Alkaloid	*Didemnum* sp. / Near Haegeumgang, Geoje in the South Sea of Korea	MeOH/CH_2Cl_2 (1:1)	-LLP, CC (Sephadex LH-20), RP FLC, RP HPLC	-HRESIMS, 1D (^{1}H, ^{13}C) and 2D NMR (COSY, HMBC)	[21]
***N*-(6-Bromo-1H-indole-3-carbonyl)-L-arginine (25)**	Alkaloid	*Didemnum* sp. / Near Haegeumgang, Geoje, Korea	MeOH/CH_2Cl_2 (1:1)	-LLP, CC (LH-20), RP FLC, RP HPLC	-HRESIMS, 1D (^{1}H, ^{13}C) and 2D NMR, (COSY, HMBC)	[21]
**(6-Bromo-1H-indol-3-yl)oxoacetamide (26)**	Alkaloid	*Didemnum* sp. / Near Haegeumgang, Geoje, Korea	MeOH/CH_2Cl_2 (1:1)	-LLP, CC (LH-20), RP FLC, RP HPLC	-HRESIMS, 1D (^{1}H, ^{13}C) and 2D NMR, (COSY, HMBC)	[21]
**2-(3,5-Diiodo-4-methoxyphenyl)ethanamine (27)**	Phenylethylamine	*Didemnum rubeum* / Chuuk Atoll	Aqueous crude extract	-CC (Sephadex LH20), RP HPLC	-LRMS, HRESIMS, 1D (^{1}H, ^{13}C) and 2D NMR (COSY, HSQC, HMBC).	[22]
**2-(3,5-Diiodo-4-methoxyphenyl)ethanaminium (28)**	Phenylethylamine	*Didemnum rubeum* / Chuuk Atoll	Aqueous crude extract	-CC (Sephadex LH20), RP HPLC	-LRMS, HRESIMS, 1D (^{1}H, ^{13}C) and 2D NMR (COSY, HSQC, HMBC).	[22]
**2-(3,5-Diiodo-4-methoxyphenyl)ethanaminium benzoate (29)**	Phenylethylamine	*Didemnum rubeum* / Chuuk Atoll	Aqueous crude extract	-CC (Sephadex LH20), RP HPLC	-LRMS, HRESIMS, 1D (^{1}H, ^{13}C) and 2D NMR (COSY, HSQC, HMBC)	[22]

**2-(3,5-Diiodo-4-methoxyphenyl)acetamide (30)**	Phenylethylamine	*Didemnum rubeum* / Chuuk Atoll	Aqueous crude extract	-CC (Sephadex LH20), RP HPLC	-LRMS, HRESIMS, 1D (^{1}H, ^{13}C) and 2D NMR (COSY, HSQC, HMBC)	[22]
***N*-[2-(3,5-Diiodo-4-methoxyphenyl)ethyl]formamide (31)**	Phenylethylamine	*Didemnum rubeum* / Chuuk Atoll	Aqueous crude extract	-CC (Sephadex LH20), RP HPLC	-LRMS, HRESIMS, 1D (^{1}H, ^{13}C) and 2D NMR (COSY, HSQC, HMBC)	[22]
***N*-[2-(3,5-Diiodo-4-methoxyphenyl)ethyl]benzamide (32)**	Phenylethylamine	*Didemnum rubeum* / Chuuk Atoll	Aqueous crude extract	-CC (Sephadex LH20), RP HPLC	-LRMS, HRESIMS, 1D (^{1}H, ^{13}C) and 2D NMR (COSY, HSQC, HMBC)	[22]
***N,N'*-Bis[2-(3,5-diiodo-4-methoxyphenyl)ethyl] ethanediamide (33)**	Phenylethylamine	*Didemnum rubeum* / Chuuk Atoll	Aqueous crude extract	-CC (Sephadex LH20), RP HPLC	-LRMS, HRESIMS, 1D (^{1}H, ^{13}C) and 2D NMR (COSY, HSQC, HMBC)	[22]
**4-(2-Aminoethyl)-2-iodophenol (34)**	Phenylethylamine	*Didemnum rubeum* / Chuuk Atoll	Aqueous crude extract	-CC (Sephadex LH20), RP HPLC	-LRMS, HRESIMS, 1D (^{1}H, ^{13}C) and 2D NMR (COSY, HSQC, HMBC)	[22]
**Minimide (35)**	Cyclic peptide / cyanobactin	*Didemnum molle* / Solomon Islands	MeOH / metagenomics	-LCMS	-LCMS, metagenomics, synthetic biology	[23]
**5α,8α-Epidioxycholest-6-en-3β-ol (36)**	Steroid	*Didemnum salary* / Andavadoaka, Madagaskar	$CHCl_3$/MeOH (1:1)	-CC (Silica gel, Sephadex LH20)	-1D NMR (^{1}H NMR)	[24]
**5α,8α-Epidioxy-24(*S*)-methylcholest-6-en-3β-ol (37)**	Steroid	*Didemnum salary* / Andavadoaka, Madagaskar	$CHCl_3$/MeOH (1:1)	-CC (Silica gel, Sephadex LH20)	-1D NMR (^{1}H NMR)	[24]
**5α,8α-Epidioxy-24(*R*)-methylcholest-6-en-3β-ol (38)**	Steroid	*Didemnum salary* / Andavadoaka, Madagaskar	$CHCl_3$/MeOH (1:1)	-CC (Silica gel, Sephadex LH20)	-1D NMR (^{1}H NMR)	[24]
**5α,8α-Epidioxy-24(*S*)-ethylcholest-6-en-3β-ol (39)**	Steroids	*Didemnum salary* / Andavadoaka, Madagaskar	$CHCl_3$/MeOH (1:1)	-CC (Silica gel, Sephadex LH20)	-1D NMR (^{1}H NMR)	[24]
**5α,8α-Epidioxy-24(*R*)-ethylcholest-6-en-3β-ol (40)**	Steroids	*Didemnum salary* / Andavadoaka, Madagaskar	$CHCl_3$/MeOH (1:1)	-CC (Silica gel, Sephadex LH20)	-1D NMR (^{1}H NMR)	[24]
**3,5-Dibromotetra-methyltyrosine (41)**	Phenylethylamine	*Didemnum* sp. / White Sands Reef, Algoa Bay, South Africa	MeOH/CH_2Cl_2	-LC-ICP-MS/ESI-MS	-1D NMR (^{1}H NMR)	[25]
**3-Iodotetramethyl-tyrosine (42)**	Phenylethylamine	*Didemnum* sp. / White Sands Reef, Algoa Bay, South Africa	MeOH/CH_2Cl_2	-LC-ICP-MS/ESI-MS	-1D NMR (^{1}H NMR)	[25]

**6-Bromotryptamine (44)**	Alkaloid / brominated indole derivative	*Didemnum candidum* / Southern Gulf of California, Mexico	MeOH	-CC (Sephadex LH-20), FLC (silica gel)	-UV, IR, MS, 1D (^{1}H, ^{13} C) and 2D NMR (COSY)	27
**2,2-Bis(6'-bromo-3'-in-doly1)ethylamine (45)**	Alkaloid / brominated indole derivative	*Didemnum candidum* / Southern Gulf of California, Mexico	MeOH	-CC (Sephadex LH-20), FLC (silica gel)	-UV, IR, MS, 1D (^{1}H, ^{13} C) 2D NMR (COSY)	27
**2,5-Bis(6'-bromo-3'-in-doly1)piperazine (46)**	Alkaloid / brominated indole derivative	*Didemnum candidum* / Southern Gulf of California Mexico	MeOH	-CC (Sephadex LH-20) and FLC (silica gel)	-UV, IR, MS, 1D (^{1}H, ^{13} C) 2D NMR (COSY)	27
**Didmolamides A and B (12 and 13)**	Cyclic peptide / cyanobactin	*Didemnum molle* / Madagascar	$CHCl_3$/MeOH (2:1)	-LLP, CC (Sephadex LH-20), HPLC	-FTIR, HR-EIMS, 1D (^{1}H, ^{13}C) and 2D NMR (COSY, HMQC, HMBC)	18
**Ascidiatrienolides A-C (47-49)**	Eicosanoids	*Didemnum candidum/* Philippines	Acetone	-VLC (silica gel), NP HPLC	-UV, IR, HRBIMS, 1D (^{1}H, ^{13}C) and 2D NMR (COSY)	28
**5′-Deoxy-3-iodotubercidin (50)**	Nucleoside	*Didemnum voeltzkowi/* Philippines	MeOH	-LLP, C18 VLC, RP HPLC	-FABMS, 1D NMR (^{1}H, ^{13}C NMR)	29
**5′-Deoxy-3-bromotubercidin (51)**	Nucleoside	*Didemnum voeltzkowi/* Philippines	MeOH	-LLP, C18 VLC, RP HPLC	-FABMS and 1D NMR (^{1}H, ^{13}C NMR)	29
**5′-Deoxytubercidin (52)**	Nucleoside	*Didemnum voeltzkowi /* Philippines	MeOH	-LLP, C18 VLC, RP HPLC	-FABMS and 1D NMR (^{1}H, ^{13}C NMR)	29
**Hydroxy phenyldienoic acid (53)**	Polyketide / phenylpolyene	*Didemnum granulatum /* Red Sea	EtOAc	-CC (Sephadex LH-20)	-UV, MS, 1D (^{1}H, ^{13}C) and 2D NMR(COSY)	30
**Enterocin (175)**	Polyketide / type II PKS	*Didemnum* sp. / Australia	70% MeOH in CH_2Cl_2	-LLPC and VLC, RP HPLC	-HRMS, IR, UV, 1D (^{1}H, ^{13}C) and 2D NMR (COSY, HMBC, NOSY)	67
**5-Deoxyenterocin (176)**	Polyketide / type II PKS	*Didemnum* sp. / Australia	70% MeOH in CH_2Cl_2	-LLPC, VLC, RP HPLC	-HRMS, IR, UV, 1D (^{1}H, ^{13}C) and 2D NMR (COSY, HMBC, NOSY)	67
**Enterocin-5-behenate (177)**	Polyketide / type II PKS	*Didemnum* sp. / Australia	70% MeOH in CH_2Cl_2	-LLPC, VLC, RP HPLC	-HRMS, IR, UV, 1D (^{1}H, ^{13}C) and 2D NMR (COSY, HMBC, NOSY)	67
**Asterubin (54)**	Guanidine derivative	*Didemnum ligulum/* São Sebastião, Brazil	MeOH	-CC (Sephadex LH-20), RP HPLC	-HRMS, 1D (^{1}H, ^{13}C) and 2D NMR (COSY, HMQC, HMBC)	31
***N,N*-Dimethyl-*O*-methylethanolamine (55)**	Ethylamine derivative	*Didemnum ligulum /* São Sebastião, Brazil	MeOH	-CC (Sephadex LH-20), RP HPLC	-HRMS, 1D (^{1}H, ^{13}C) and 2D NMR (COSY, HMQC, HMBC)	31
